# TOMOC: a topology-driven multi-objective evolutionary framework for robust overlapping protein complex detection in noisy PPI networks

**DOI:** 10.1186/s12859-026-06603-z

**Published:** 2026-08-18

**Authors:** Mustafa Abbas, David Broneske, Gunter Saake

**Affiliations:** 1 Databases and Software Engineering, Otto-von-Guericke-University, Magdeburg, Germany; 2 German Centre for Higher Education Research and Science Studies, Hanover, Germany

**Keywords:** Protein–protein interaction networks, Protein complex detection, Overlapping complexes, Multi-objective evolutionary optimization, Network topology, Evolutionary algorithms

## Abstract

****Background**:**

Protein complexes constitute fundamental functional modules within cells and play a crucial role in regulating many biological processes. Detecting protein complexes from protein-protein interaction (PPI) networks has therefore become a central problem in computational systems biology. However, many existing computational approaches struggle to accurately identify overlapping complexes where proteins participate in multiple functional modules simultaneously. In addition, large-scale PPI networks are inherently noisy and incomplete due to experimental limitations, which significantly affects the reliability of complex detection methods.

****Results**:**

In this work, we propose TOMOC, a topology-driven multi-objective evolutionary framework for robust detection of overlapping protein complexes in noisy PPI networks. The novelty of TOMOC lies in the integration of a topology-driven bi-objective formulation, an edge-based evolutionary representation that naturally supports overlapping memberships, and a topology-aware structural refinement mechanism within a unified framework for protein complex detection in noisy PPI networks. The proposed framework introduces an edge-based evolutionary representation that models candidate solutions at the interaction level, allowing overlapping memberships to emerge naturally during decoding. It further optimizes two complementary structural objectives by minimizing average conductance and triangle-density loss, enabling the algorithm to balance boundary quality and internal structural density. In addition, a topology-aware structural overlap refinement (SOR) operator is designed to improve structural coherence and robustness against noisy interactions through boundary-aware repair, triangle-closure expansion, and triangle-support pruning. Extensive experiments conducted on three benchmark PPI networks (Yeast-D1, Yeast-D2, and Collins) demonstrate that TOMOC achieves competitive performance compared with several state-of-the-art methods in terms of precision, recall, and F1-score.

****Conclusions**:**

The proposed TOMOC framework provides an effective and scalable topology-driven approach for detecting overlapping protein complexes directly from PPI network topology. By integrating multi-objective evolutionary optimization with topology-aware refinement mechanisms, TOMOC effectively captures the structural characteristics of protein complexes and demonstrates strong robustness when applied to large and noisy biological interaction networks.

## Introduction

Proteins are fundamental functional units of living cells and participate in diverse biological processes, including signal transduction, metabolism, and cellular regulation. Rather than functioning independently, proteins interact to form coordinated molecular assemblies known as protein complexes. Protein-protein interaction (PPI) networks provide a systems-level representation of these interactions and are widely used to investigate cellular organization and functional relationships among proteins [1–3]. Advances in high-throughput experimental technologies, particularly yeast two-hybrid assays and affinity purification coupled with mass spectrometry, have enabled the large-scale reconstruction of PPI networks and created new opportunities for computational analysis of cellular systems [4, 5].

Accurate detection of protein complexes from PPI networks nevertheless remains challenging. Experimentally derived networks are typically incomplete and noisy, containing both spurious interactions and missing links [6–8]. Furthermore, many traditional clustering methods assign each protein to a single cluster, although proteins may participate in multiple functional assemblies. This biological reuse of proteins gives rise to overlapping complexes that cannot be adequately represented by disjoint network partitions [9, 10]. Effective detection methods should therefore support overlapping memberships while remaining resilient to uncertainty in the underlying interaction network.

Numerous computational approaches have been proposed for protein-complex detection, including graph-clustering, density-based, and evolutionary methods. Classical algorithms such as MCL [11], MCODE [12], and CPM [13] identify densely connected network regions, but they may produce disjoint clusters, depend strongly on density-related parameters, or require additional processing to represent overlapping memberships. More recent methods explicitly address overlapping complex detection, yet their performance may remain sensitive to network noise, parameter selection, or the availability of auxiliary biological annotations. These limitations motivate the development of topology-driven approaches that can identify overlapping structures without requiring external biological information during optimization.

Protein-complex detection can naturally be formulated as a multi-objective optimization problem because different structural properties may favor different network partitions. A biologically plausible complex should exhibit strong internal connectivity while remaining sufficiently separated from the surrounding network. Optimizing either property alone may produce incomplete, fragmented, or excessively broad modules. Jointly considering complementary structural objectives therefore provides a principled mechanism for exploring alternative trade-offs and identifying nondominated candidate solutions.

To address these challenges, we propose TOMOC, a topology-driven multi-objective evolutionary framework for detecting overlapping protein complexes in PPI networks. TOMOC operates exclusively on network topology and does not use functional annotations during optimization. It employs an edge-based representation that captures interaction-level structure and enables overlapping protein memberships during decoding. The framework jointly optimizes average conductance and triangle-density loss to balance boundary separation and internal cohesion. It further incorporates a topology-aware Structural Overlap Refinement (SOR) mechanism that refines decoded candidate complexes by preserving structurally supported memberships and reducing weak noise-induced associations.

**Fig. 1:**
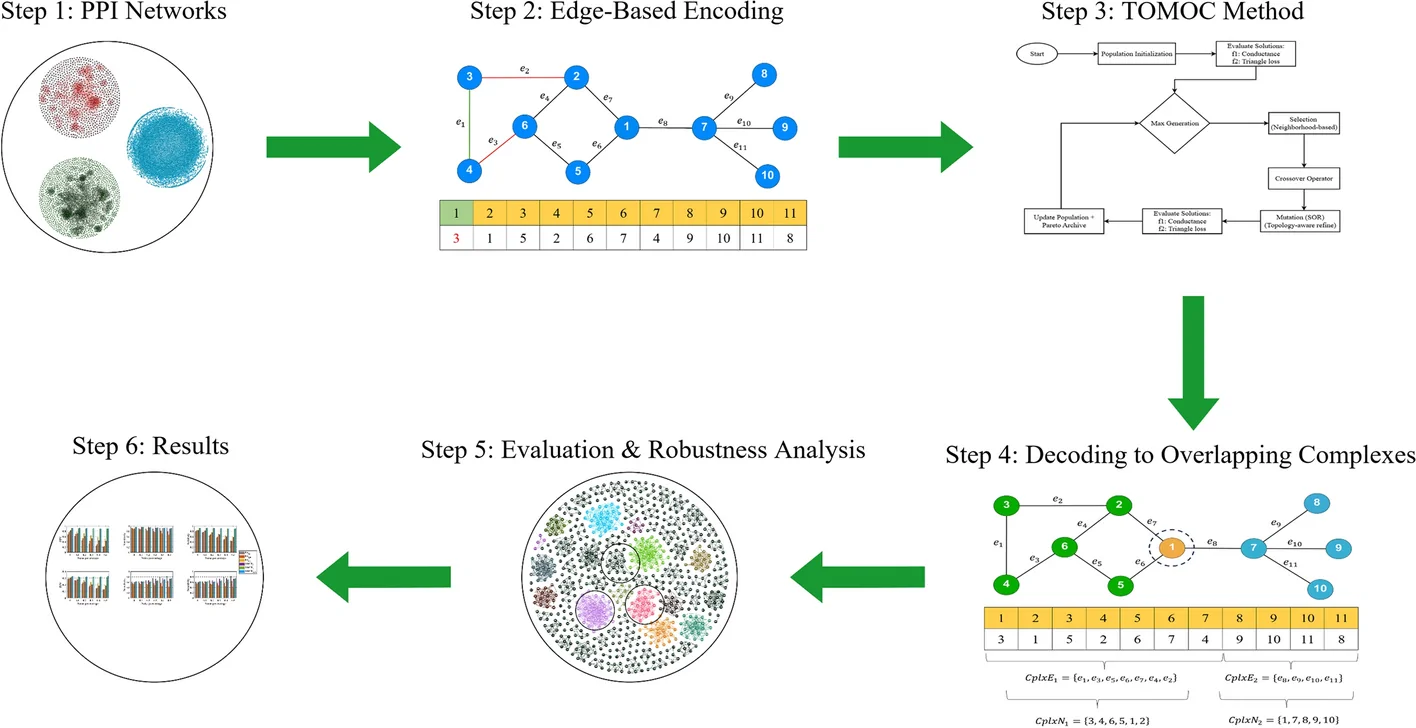
Overview of the proposed TOMOC framework. A PPI network is transformed into an edge-based chromosome representation and processed using multi-objective evolutionary optimization. Candidate solutions are decoded into overlapping protein complexes and refined using the topology-aware SOR mechanism. The resulting complexes are subsequently evaluated using structural benchmark metrics and, independently, post-hoc Gene Ontology-based biological validation

The main contributions of this work are fourfold. First, we formulate overlapping protein-complex detection as a bi-objective optimization problem that jointly considers complex separation and internal connectivity. Second, we develop an edge-based evolutionary framework with topology-aware decoding and structural overlap refinement to generate overlapping candidate complexes. Third, we conduct an extensive evaluation that includes comparisons with modern overlapping protein-complex detection methods and controlled interaction insertion and deletion experiments for assessing robustness to network noise. Fourth, we perform independent post-hoc biological validation using gene ontology semantic coherence, enrichment analysis, and representative biological case studies. gene ontology annotations are used exclusively for validation and are not involved in representation, objective evaluation, evolutionary search, or Pareto-solution generation. An overview of the complete framework and its independent evaluation stages is presented in Fig. 1.

## Related work

Computational methods for protein-complex detection can broadly be categorized into classical graph-clustering approaches, methods explicitly designed to recover overlapping complexes, and evolutionary or metaheuristic frameworks. Early graph-clustering methods generally searched for densely connected subnetworks. MCL [11], for example, identifies clusters by simulating random walks through the interaction network, whereas MCODE [12] detects locally dense regions through vertex weighting, seed selection, and cluster expansion. Clique-based approaches, including CPM [13] and CFinder [14], identify communities formed by adjacent cliques and can therefore represent overlapping memberships. Although these methods established important foundations for network-based protein-complex detection, their outputs may be sensitive to seed-selection strategies, density thresholds, or clique-size parameters, particularly when applied to sparse and noisy PPI networks.

Several subsequent methods were developed specifically to identify overlapping protein complexes. ClusterONE [15] expands locally cohesive protein groups while allowing individual proteins to participate in multiple complexes. Other topology-driven methods, including PEWCC, EGCPI, GMFTP, and LADOC, combine edge weighting, seed expansion, local structural information, or adaptive density criteria to recover overlapping modules [16–19]. More recent approaches, such as CACO, AdaPPI, and FMvPCI, further improve overlapping-complex detection through adaptive clustering, refined topological scoring, or multi-view structural information [20–22]. Related topology-based methods, including EWCA [23], entropy-driven clustering [24], and DOPCC [25], also exploit local connectivity patterns to improve complex recovery. Despite their effectiveness, these approaches may remain dependent on predefined similarity functions, seed-selection procedures, or method-specific structural thresholds.

Evolutionary and metaheuristic algorithms provide an alternative mechanism for exploring the large combinatorial search space associated with network clustering. Single-objective evolutionary approaches have optimized structural criteria such as community score, expansion, ratio cut, normalized cut, internal density, modularity, and related community-quality measures [26, 27]. Multi-objective methods instead preserve trade-offs between complementary structural properties rather than combining them into a single aggregate objective. Bandyopadhyay et al. [28] proposed a multi-objective community-detection approach, denoted as MOCD$_1$ in this study. Attea et al. [29] subsequently proposed an improved multi-objective strategy, denoted as MOCD$_2$. These two methods are included as multi-objective evolutionary baselines in our experimental evaluation. Other evolutionary approaches applied to network or protein-complex detection include PPI-GA [30] and MOEPGA [31].

Although evolutionary methods provide flexible search mechanisms, many existing formulations rely on node-based encodings or primarily generate disjoint partitions. Consequently, overlapping memberships may require additional decoding rules, post-processing procedures, or specialized operators. Furthermore, robustness under controlled interaction insertion and deletion has not always been examined explicitly, despite the incompleteness and noise commonly associated with experimentally derived PPI networks.

The existing literature therefore reveals three closely related challenges. First, overlapping membership should emerge naturally from the solution representation and decoding process rather than being introduced only after clustering. Second, the optimization procedure should remain applicable when functional annotations are incomplete or unavailable. Third, complementary structural properties should be optimized jointly and evaluated systematically under controlled network perturbations. TOMOC addresses these requirements through an edge-based representation, a bi-objective evolutionary search based on average conductance and triangle-density loss, and a topology-aware Structural Overlap Refinement operator. Unlike methods that incorporate biological annotations during complex detection, TOMOC uses only PPI-network topology throughout the optimization process. gene ontology information is employed exclusively afterward as an independent post-hoc validation of the functional coherence and biological relevance of the detected complexes.

## Preliminaries

This section introduces the fundamental graph-theoretic concepts required to describe the proposed framework.

A PPI network is modeled as an undirected graph *G=(V,E)*, where $V=\{v_1, v_2, \dots , v_n\}$ represents the set of proteins and *E* denotes the set of pairwise interactions. Each vertex corresponds to a protein, and an edge $(v_i, v_j)\in E$ indicates an interaction between proteins $P_i$ and $P_j$. The degree of a vertex $v_i$ is defined as the number of its adjacent interactions.

For computational convenience, the network can be represented by an adjacency matrix $\textbf{A}=[a_{ij}]^{n\times n}$, where $a_{ij}=1$ if $(v_i,v_j)\in E$ and 0 otherwise. This representation provides a compact description of the network topology used in the proposed method.

Let $\mathcal {C}=\{C_1, C_2, \dots , C_K\}$ denote a set of detected protein complexes, where each $C_k \subseteq V$. Unlike classical clustering formulations, this definition allows proteins to belong to multiple complexes, enabling overlapping memberships.

Local neighborhood information plays a key role in the proposed edge-based representation. For a vertex $v_i \in V$, its neighborhood is defined as

1 $$\begin{aligned} \mathcal {N}(v_i)=\{v_j\in V \mid (v_i,v_j)\in E\} \end{aligned}$$

Similarly, for an edge $e_i=(u,v)\in E$, the edge neighborhood is defined as

2 $$\begin{aligned} \mathcal {N}_E(e_i)=\{e_j\in E \mid e_j\cap e_i\ne \emptyset \} \end{aligned}$$

Two edges are considered adjacent if they share at least one endpoint. These definitions form the basis of the edge-based encoding and decoding procedures described in the following section.

## Method

Detecting protein complexes in PPI networks requires balancing multiple, often conflicting, structural properties. High-quality complexes should exhibit strong internal cohesion, clear boundary separation, and topological consistency, while allowing overlapping memberships that reflect multifunctional proteins. Optimizing these properties within a single objective often leads to either fragmented or overly merged structures. Therefore, a multi-objective formulation is essential to explicitly capture and balance these competing criteria.

To address these challenges, we propose TOMOC (Topology-Driven Multiobjective Optimization for Overlapping Complexes), an evolutionary framework for topology-based overlapping protein complex detection. Unlike many existing methods that rely on external biological annotations or post-processing strategies, TOMOC operates directly on network topology and allows overlapping structures to emerge naturally during the search process.

TOMOC is built upon a decomposition-based multi-objective evolutionary strategy inspired by MOEA/D [32]. It simultaneously optimizes two complementary objectives: (i) average conductance, which promotes well-separated complexes, and (ii) triangle-density loss, which encourages dense and locally cohesive structures. These objectives capture complementary aspects of community quality and guide the search toward structurally robust overlapping complexes.

A central component of TOMOC is its edge-based representation, where each individual encodes relationships among network edges rather than assigning nodes directly to clusters. Through a deterministic decoding process, edge groups are transformed into node-based complexes. Since edges incident to the same protein may belong to different groups, overlapping memberships arise naturally without explicit constraints. Moreover, the number of complexes is not predefined but emerges adaptively during the evolutionary process.

To enhance structural coherence, TOMOC incorporates a topology-aware mutation operator termed Structural Overlap Refinement (SOR). This operator performs localized adjustments guided by network topology, reinforcing well-supported intra-complex structures while suppressing weak or noise-induced memberships. As a result, it improves robustness and stability in noisy PPI networks.

### Objective formulation

The effectiveness of a multi-objective evolutionary framework depends on objective functions that accurately reflect the structural properties of biologically meaningful protein complexes. In PPI networks, such complexes are typically characterized by two complementary topological properties: strong internal cohesion with limited boundary leakage, and high local transitivity indicating densely interconnected structures.

To capture these principles, TOMOC optimizes two complementary and inherently conflicting objectives: average conductance and triangle-density loss. Both objectives are defined solely in terms of network topology and do not rely on external biological annotations.

#### Objective 1: average conductance minimization

For a complex $C_k \subseteq V$, let $m_{\text {in}}(C_k)$ denote the number of internal edges, $\text {vol}(C_k)$ the sum of vertex degrees, and $\text {cut}(C_k)$ the number of boundary edges. The conductance of $C_k$ is defined as:

3 $$\begin{aligned} \phi (C_k) = \frac{\text {cut}(C_k)}{\min (\text {vol}(C_k), \text {vol}(V) - \text {vol}(C_k)) + \varepsilon } \end{aligned}$$

where *\varepsilon * is a small constant to avoid division by zero. Lower conductance values indicate better-separated and structurally coherent complexes.

For a candidate solution $\mathcal {C} = {C_1, \dots , C_K}$, the objective is:

4 $$\begin{aligned} \min f_1(\mathcal {C}) = \frac{1}{K_v} \sum _{k=1}^{K_v} \phi (C_k), \end{aligned}$$

where $K_v$ is the number of valid complexes with $|C_k| \ge 3$.

#### Objective 2: triangle-density loss minimization

To promote internal cohesion, the second objective is based on triangle density. For a complex $C_k$ of size $s_k$, the number of triangles is computed as:

5 $$\begin{aligned} T(C_k) = \frac{\text {trace}(\textbf{A}_{C_k}^3)}{6}, \end{aligned}$$

where $\textbf{A}_{C_k}$ is the induced adjacency matrix.

The normalized triangle density is defined as:

6 $$\begin{aligned} \text {TriDen}(C_k) = \frac{T(C_k)}{\left( {\begin{array}{c}s_k\\ 3\end{array}}\right) + \varepsilon } \end{aligned}$$

To maintain a minimization formulation, the triangle-density loss is defined as:

7 $$\begin{aligned} \text {TriLoss}(C_k) = 1 - \text {TriDen}(C_k). \end{aligned}$$

The second objective is then:

8 $$\begin{aligned} \min f_2(\mathcal {C}) = \frac{1}{K_v} \sum _{k=1}^{K_v} \text {TriLoss}(C_k). \end{aligned}$$

#### Complementarity of objectives

The two objectives capture complementary aspects of community structure. Minimizing conductance promotes clear boundary separation, whereas minimizing triangle-density loss encourages dense and transitive subgraphs. These objectives are inherently conflicting: emphasizing boundary separation may lead to loosely connected clusters, while prioritizing triangle density may result in overly compact or fragmented structures.

By jointly optimizing $f_1$ and $f_2$ within a decomposition-based multi-objective framework, TOMOC enables the discovery of Pareto-optimal solutions that balance boundary consistency and internal cohesion without imposing predefined cluster numbers or explicit overlap constraints.

### Edge-based representation and decoding

A key component of TOMOC is its edge-based solution representation, which fundamentally differs from traditional node-partitioning approaches. Instead of assigning proteins directly to clusters, candidate solutions are defined at the interaction level. This design is motivated by the observation that biological functionality in PPI networks emerges from interaction patterns rather than isolated nodes. By operating on edges, the representation naturally supports overlapping memberships, since a protein may participate in multiple interaction groups.

Each individual (chromosome) is represented as a vector:

9 $$\begin{aligned} I=(I_1,I_2,\dots ,I_m), \end{aligned}$$

where each gene $I_i$ corresponds uniquely to an edge $e_i \in E$.

For each edge $e_i = (u,v)$, the admissible values of gene $I_i$ are restricted to edges adjacent to $e_i$, defined as:

10 $$\begin{aligned} \mathcal {N}_E(e_i)={e_j\in E \mid e_i\cap e_j\ne \emptyset }. \end{aligned}$$

Each gene therefore encodes a successor relation by redirecting $e_i$ to one of its neighboring edges. This induces a directed functional graph over the edge set, where each edge selects exactly one adjacent edge.

Unlike node-based encodings, this representation does not explicitly assign nodes to clusters. Instead, it defines connectivity relationships among edges, from which node-based complexes are derived during decoding. As a result, overlapping memberships arise naturally when edges incident to the same protein belong to different edge groups.

#### Extraction of edge complexes

Given a chromosome *I*, the decoding process first constructs edge-level components. Each gene induces a successor relation over edges, forming a directed functional graph where each edge $e_i$ points to exactly one adjacent edge $e_{I_i}$.

Edge complexes are extracted by traversing these successor links. Starting from an unvisited edge $e_i$, successive redirections are followed:

$$ e_i \rightarrow e_{I_i} \rightarrow e_{I_{I_i}} \rightarrow \dots $$

until a previously visited edge is encountered. All traversed edges are grouped into the same edge complex. The process is repeated until all edges are assigned.

Formally, this procedure partitions *E* into disjoint edge complexes:

$$ \mathcal {C}_E = \{C^E_1, C^E_2, \dots , C^E_{K_E}\}. $$

Each edge complex represents a structurally connected interaction pattern induced by the chromosome. The number of edge complexes $K_E$ is implicitly determined by the encoding and requires no prior specification.

#### Conversion to node-based overlapping complexes

In the second stage, each edge complex $C^E_k$ is transformed into a node-based complex by aggregating the endpoints of its constituent edges:

11 $$\begin{aligned} C_k = { v \in V \mid \exists (u,v) \in C^E_k }. \end{aligned}$$

Because distinct edge complexes may share nodes through common interaction endpoints, the resulting node complexes are inherently overlapping.

Importantly, this overlap is not imposed through external constraints or post-processing procedures. Instead, it emerges directly from the underlying edge-based representation. A protein participates in multiple complexes when its incident edges belong to different edge components, naturally reflecting its multifunctional role in the network.

**Fig. 2:**
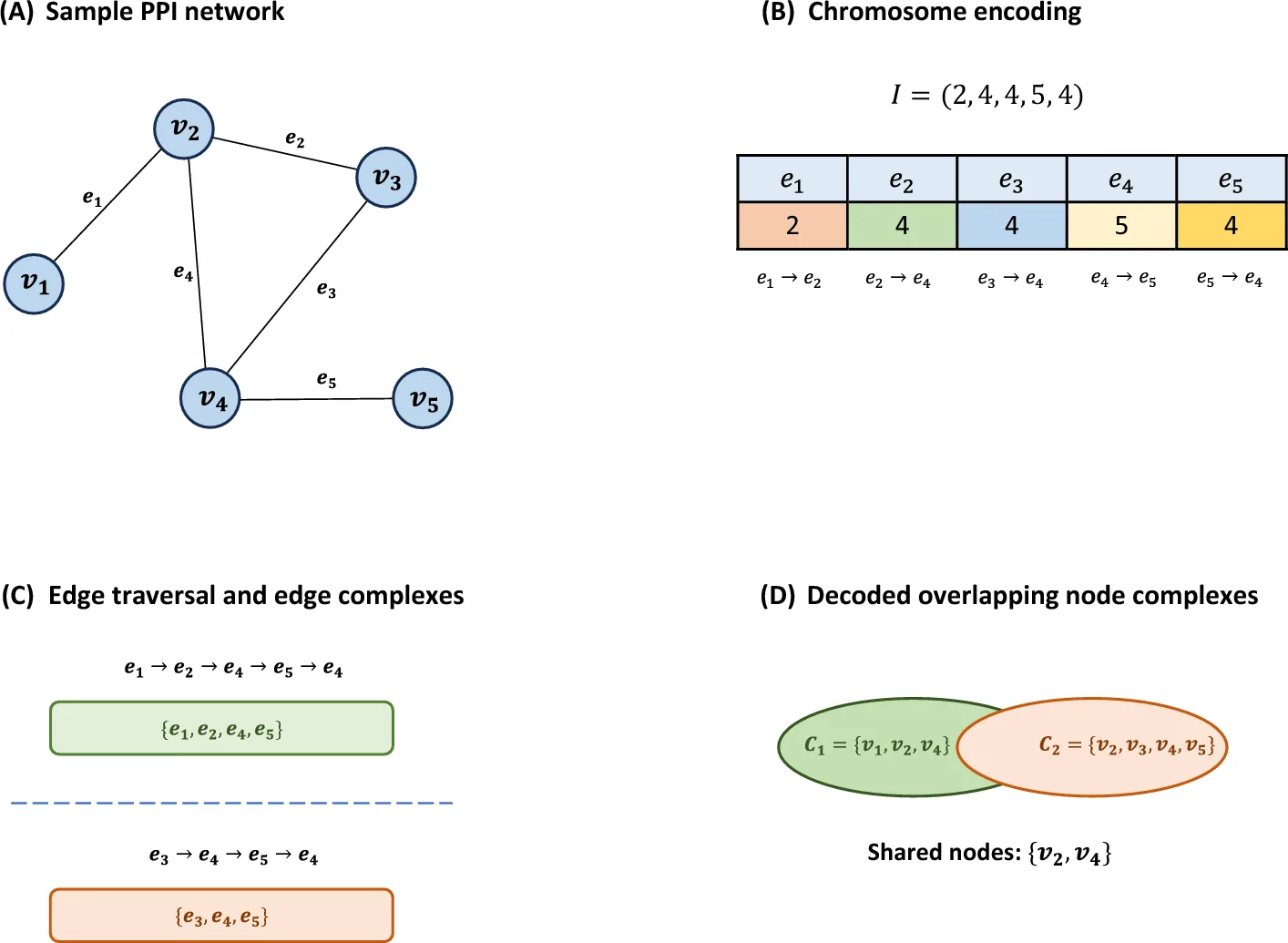
Step-by-step illustrative example of the proposed edge-based encoding and decoding process. **A** A sample PPI network with five proteins and five labeled interactions. **B** Example chromosome encoding, where each gene stores the index of an adjacent successor edge. **C** Successor traversal induces edge-level components. **D** Decoding these edge components into node sets naturally yields overlapping protein complexes through shared vertices

To make the proposed encoding and decoding process easier to follow, Fig. 2 provides a step-by-step illustrative example based on a small PPI network. Starting from a sample graph with five proteins and five labeled interactions, each edge is encoded as a gene that points to one of its adjacent edges. These successor relations induce edge-level components, which are then decoded into node-based complexes by collecting the endpoints of the corresponding edges. Because different edge components may share vertices, overlapping memberships arise naturally during decoding.

The detailed procedure for decoding the edge-based chromosome into overlapping node-based complexes is provided in Supplementary Algorithm S1.

### Recombination operator

To promote structural exploration while preserving topological feasibility, TOMOC employs a uniform recombination operator tailored to the edge-based representation. The operator combines linkage patterns from two parent chromosomes to generate a valid offspring.

Let $\mathcal {I}^{(1)}$ and $\mathcal {I}^{(2)}$ denote two parent chromosomes:

12 $$\begin{aligned} \mathcal {I}^{(p)} = (\mathcal {I}^{(p)}_1, \dots , \mathcal {I}^{(p)}_m), \quad p \in {1,2}. \end{aligned}$$

The offspring $\mathcal {I}'$ is generated as:

13 $$\begin{aligned} \mathcal {I}'_j = {\left\{ \begin{array}{ll} \mathcal {I}^{(1)}_j, & \text {if } \chi _j < 0.5, \\ \mathcal {I}^{(2)}_j, & \text {otherwise}, \end{array}\right. } \quad j = 1,\dots ,m, \end{aligned}$$

where $\chi _j \sim \mathcal {U}(0,1)$ is an independent uniform random variable for each gene position.

Since each gene encodes a successor relation to an adjacent edge, copying genes from either parent preserves feasibility without requiring repair. Recombination therefore mixes local edge-linkage structures, enabling the propagation of coherent substructures across generations.

Because the encoding operates at the interaction level, the resulting node complexes are influenced indirectly through the decoded edge components, allowing overlapping structures to evolve naturally.

### Topology-aware mutation: structural overlap refinement (SOR)

While recombination promotes global structural mixing, refining overlapping complexes requires carefully guided local adjustments. Purely random mutation can easily disrupt well-formed edge structures and weaken internal cohesion. To address this limitation, we introduce a topology-aware mutation operator, *Structural Overlap Refinement (SOR)*, which performs targeted modifications to improve boundary consistency and triangle-supported cohesion within overlapping complexes.

In contrast to conventional mutation operators that randomly perturb successor assignments, SOR operates directly on the decoded structure. It applies localized, topology-driven corrections that are explicitly aligned with the structural objectives of TOMOC, enabling more stable and meaningful refinements.

Given a decoded overlapping structure $\mathcal {C} = {C_1, C_2, \dots , C_K}$, SOR iteratively adjusts memberships by examining both boundary connectivity and triangle-based support within each complex.

For a protein $P_i \in C_k$, its structural consistency is first evaluated through its internal and external connectivity. The internal degree is defined as

14 $$\begin{aligned} d_{\text {in}}(P_i, C_k) = \sum _{P_j \in C_k} A_{ij}, \end{aligned}$$

while the external connectivity is given by

15 $$\begin{aligned} d_{\text {cut}}(P_i, C_k) = \sum _{P_j \notin C_k} A_{ij}. \end{aligned}$$

If the internal support is insufficient relative to external connections, i.e.,

16 $$\begin{aligned} d_{\text {in}}(P_i, C_k) < \tau \cdot d_{\text {cut}}(P_i, C_k), \end{aligned}$$

where $\tau \in (0,1)$ controls tolerance, the membership is considered boundary-inconsistent and is removed. This operation suppresses excessive cross-boundary interactions and contributes to lowering the conductance of the complex.

Beyond boundary consistency, SOR reinforces cohesive structure by incorporating triangle-based expansion. For a protein $P_i \notin C_k$, the number of triangles it forms with members of $C_k$ is computed as

17 $$\begin{aligned} T(P_i, C_k) = \left| \left\{ (P_j, P_l) \in C_k : A_{ij}=1, A_{il}=1, A_{jl}=1 \right\} \right| . \end{aligned}$$

If $T(P_i, C_k) \ge \gamma$, where *γ * is a small integer threshold, the protein is added to $C_k$, promoting densely interconnected substructures.

To eliminate weakly supported members, SOR further evaluates the internal triangle participation of proteins already assigned to the complex:

18 $$\begin{aligned} T_{\text {in}}(P_i, C_k) = \left| \left\{ (P_j, P_l) \in C_k : A_{ij}=1, A_{il}=1, A_{jl}=1 \right\} \right| . \end{aligned}$$

If $T_{\text {in}}(P_i, C_k) < \delta$, where *δ * is a minimum triangle-support threshold, the protein is removed. This prevents loosely connected or noise-induced memberships from persisting in the complex.

The complete procedure of the SOR operator is provided in Supplementary Algorithm S2.

## Experimental design

This section describes the datasets, reference complex sets, evaluation measures, and experimental settings used to assess TOMOC. The evaluation includes comparisons with representative heuristic-based, evolutionary, and overlapping protein-complex detection methods, together with robustness analysis under controlled perturbations of the PPI networks.

### Datasets and reference complexes

The experimental evaluation was conducted using two complementary groups of PPI benchmark settings, all derived from *Saccharomyces cerevisiae*. The first group was used for the primary performance evaluation, comparison with evolutionary baselines, and robustness analysis. The second group was used for the additional comparison with representative overlapping protein-complex detection methods and for the independent GO-based biological validation.

For the primary experiments, we considered the Yeast-D1, Yeast-D2, and Collins PPI networks. Yeast-D1 is derived from the high-confidence yeast interaction map reported by Gavin et al. [33] and was subsequently filtered following the procedure described by Zaki et al. [34]. The resulting network contains 990 proteins and 4,687 interactions.

Yeast-D2 is an integrated yeast PPI network constructed from multiple experimental sources and processed using the same filtering procedure [34]. It contains 1,443 proteins and 6,993 interactions. Compared with Yeast-D1, Yeast-D2 provides a larger interaction space and a reference set containing a greater degree of complex overlap.

The Collins network was obtained from curated yeast interaction data available through BioGRID [35]. It contains 1,004 proteins and 8,319 interactions and is the most densely connected of the three primary PPI networks.

For the primary performance evaluation, Yeast-D1 and Yeast-D2 were assessed using the Complex-D1 and Complex-D2 reference sets, respectively. Both reference sets are derived from the MIPS protein-complex catalogue [36]. The Collins network was primarily evaluated using the CYC2008 curated yeast complex set [37]. The corresponding evaluation settings contained 78 reference complexes for Yeast-D1, 150 for Yeast-D2, and 261 for Collins-CYC2008.

The MIPS reference complex set was additionally used with the Collins network in the robustness and matching-threshold analyses. Accordingly, Collins-CYC2008 and Collins-MIPS denote two evaluation settings based on the same underlying Collins PPI network but using different reference complex sets.

For the comparison with representative overlapping protein-complex detection methods, we additionally considered the Gavin, Collins, and Krogan yeast PPI networks [4, 33]. The processed PPI network files and benchmark mappings used in this comparison are identical to those used in the comparative evaluation reported in the FMvPCI study [22]. All three networks were evaluated against the CYC2008 reference complex set using the overlap-score protocol described in  “Evaluation protocols” Section. The same three networks were also used for the independent post-hoc GO-based biological validation.

### Evaluation protocols

Two complex-level matching protocols were used in this study. The primary benchmark experiments employed the Jaccard coefficient, whereas the additional comparison with representative overlapping protein-complex detection methods followed the overlap-score protocol adopted in the corresponding published comparative evaluation. The two matching criteria were applied in separate experimental settings and were not treated as interchangeable measures.

Let $\mathcal {C}=\{C_1,C_2,\ldots ,C_K\}$ denote the set of protein complexes predicted by TOMOC, and let $\mathcal {C}^{*} =\{C_1^{*},C_2^{*},\ldots ,C_{K^{*}}^{*}\}$ denote the corresponding reference complex set.

#### Jaccard-based evaluation

For the Yeast-D1, Yeast-D2, Collins-CYC2008, and Collins-MIPS evaluation settings, the similarity between a predicted complex $C_j\in \mathcal {C}$ and a reference complex $C_i^{*}\in \mathcal {C}^{*}$ was measured using the Jaccard coefficient:

19 $$\begin{aligned} J\!\left( C_i^{*},C_j\right) = \frac{ \left| C_i^{*}\cap C_j\right| }{ \left| C_i^{*}\cup C_j\right| } \end{aligned}$$

A predicted-reference complex pair was considered matched when

20 $$\begin{aligned} J\!\left( C_i^{*},C_j\right) \ge OS \end{aligned}$$

where the matching threshold was set to *OS=0.20*, unless otherwise stated.

Based on this criterion, complex-level Recall was defined as

21 $$\begin{aligned} \textrm{Recall} = \frac{ \left| \left\{ C_i^{*}\in \mathcal {C}^{*} \;|\; \exists \,C_j\in \mathcal {C} \text { such that } J\!\left( C_i^{*},C_j\right) \ge OS \right\} \right| }{ \left| \mathcal {C}^{*}\right| } \end{aligned}$$

Complex-level Precision was defined as

22 $$\begin{aligned} \textrm{Precision} = \frac{ \left| \left\{ C_j\in \mathcal {C} \;|\; \exists \,C_i^{*}\in \mathcal {C}^{*} \text { such that } J\!\left( C_i^{*},C_j\right) \ge OS \right\} \right| }{ \left| \mathcal {C}\right| } \end{aligned}$$

The F1-score was then calculated as

23 $$\begin{aligned} \text {F1-score} = 2 \times \frac{Precision \times Recall}{Precision + Recall} \end{aligned}$$

Recall measures the proportion of reference complexes recovered by the predicted complex set, whereas Precision measures the proportion of predicted complexes that match at least one reference complex. The F1-score summarizes the balance between these two measures. This Jaccard-based protocol was used for the primary performance evaluation, the comparison with evolutionary baselines, the matching-threshold analysis, and the network-perturbation experiments.

#### Overlap-score-based evaluation

For the comparison with representative overlapping protein-complex detection methods on the Gavin, Collins, and Krogan PPI networks, we followed the overlap-score protocol used in the corresponding published comparative evaluation. The overlap score between a predicted complex $C_j\in \mathcal {C}$ and a reference complex $C_i^{*}\in \mathcal {C}^{*}$ was calculated as

24 $$\begin{aligned} \textrm{OS}\!\left( C_i^{*},C_j\right) = \frac{ \left| C_i^{*}\cap C_j\right| ^{2} }{ \left| C_i^{*}\right| \left| C_j\right| } \end{aligned}$$

A predicted-reference complex pair was considered matched when

25 $$\begin{aligned} \textrm{OS}\!\left( C_i^{*},C_j\right) \ge OS \end{aligned}$$

where the matching threshold was set to *OS=0.20*

For this protocol, Precision, Recall, and F1-score were calculated using the same complex-level counting definitions given in Eqs. (21)–(23), with the Jaccard matching condition replaced by the overlap-score matching condition in Eq. (25). This overlap-score-based protocol was used exclusively for the comparison with representative overlapping protein-complex detection methods on the Gavin, Collins, and Krogan networks.

### Experimental settings

The TOMOC population size was set to *μ =100*, and each run was executed for 100 generations, corresponding to 10,000 fitness evaluations. The recombination parameter was set to $p_c=0.8$, while the Structural Overlap Refinement procedure was applied with probability $p_m=0.2$. All TOMOC experiments were repeated over 10 independent runs for each dataset to account for stochastic variability.

### Independent go-based validation protocol

To provide an independent biological assessment of the complexes detected by TOMOC, we performed two complementary post-hoc analyses using gene ontology (GO) annotations: semantic-coherence analysis and GO enrichment analysis. These analyses were conducted on the Gavin, Collins, and Krogan evaluation settings. GO annotations for *Saccharomyces cerevisiae* were obtained from the Saccharomyces Genome Database (SGD) [38].

For each independent run, the representative solution was the solution with the highest F1-score selected from the final nondominated archive. The complexes obtained from these selected run-level solutions were subsequently evaluated using the following two biological-validation procedures.

GO-term semantic similarity was calculated using the Wang method [39]. For two proteins $P_a$ and $P_b$, let $\mathcal {T}_a$ and $\mathcal {T}_b$ denote their respective sets of GO terms. The term-level Wang similarities were aggregated using the Best Match Average (BMA) strategy:

26 $$\begin{aligned} \begin{aligned} \operatorname {Sim}_{\textrm{BMA}}(P_a,P_b) = \frac{1}{2} \Bigg [&\frac{1}{|\mathcal {T}_a|} \sum _{t_a\in \mathcal {T}_a} \max _{t_b\in \mathcal {T}_b} \operatorname {Sim}_{\textrm{Wang}}(t_a,t_b) + \frac{1}{|\mathcal {T}_b|} \sum _{t_b\in \mathcal {T}_b} \max _{t_a\in \mathcal {T}_a} \operatorname {Sim}_{\textrm{Wang}}(t_a,t_b) \Bigg ] \end{aligned} \end{aligned}$$

For a predicted complex $C_k$ containing $n_k$ GO-annotated proteins, its semantic coherence was calculated as the mean pairwise BMA similarity among its annotated members:

27 $$\begin{aligned} \operatorname {Coherence}(C_k) = \frac{2}{n_k(n_k-1)} \sum _{a=1}^{n_k-1} \sum _{b=a+1}^{n_k} \operatorname {Sim}_{\textrm{BMA}}(P_a,P_b) \end{aligned}$$

Only predicted complexes containing at least two GO-annotated proteins were included in the semantic-coherence analysis. For each evaluated complex, the observed coherence was compared with the coherence values obtained from 1000 size-matched random protein sets sampled from the corresponding PPI network.

A one-sided empirical *p*-value was calculated as the proportion of random sets whose coherence was greater than or equal to the observed coherence. The resulting *p*-values were adjusted within each run using the Benjamini-Hochberg procedure [40]. A predicted complex was considered significantly coherent when its adjusted value satisfied *q<0.05* and its observed coherence was greater than the corresponding random expectation.

For each run, annotation coverage, mean observed coherence, mean random coherence, the difference between the observed and random values, and the proportion of significantly coherent complexes were calculated. The final results were reported as the mean and standard deviation across the 10 independent runs.

GO enrichment was subsequently evaluated independently within the Biological Process (BP), Molecular Function (MF), and Cellular Component (CC) domains. For each domain, proteins in the corresponding PPI network with available annotations were used as the domain-specific background.

Predicted complexes containing fewer than three proteins were excluded from the enrichment analysis. A complex was evaluated within a GO domain only when at least three of its proteins had annotations in that domain. GO terms supported by fewer than two proteins in the predicted complex, occurring in fewer than three proteins in the domain-specific background, or covering more than 50% of the background were excluded.

For each eligible term, enrichment was assessed using the upper-tail hypergeometric test. The resulting *p*-values were adjusted separately for each predicted complex and GO domain using the Benjamini-Hochberg procedure. A complex was considered significantly enriched in a GO domain when at least one tested term satisfied *q<0.05*.

The proportions of evaluable complexes exhibiting significant enrichment in BP, MF, CC, and at least one of the three domains were calculated separately for each run. The final enrichment rates were reported as the mean and standard deviation across the 10 independent runs.

Finally, five illustrative cases were selected from significantly enriched TOMOC predictions to represent distinct, well-characterized yeast protein assemblies across the Collins, Gavin, and Krogan networks. All selected predictions matched a CYC2008 reference complex using the overlap-score criterion with *OS=0.20*.

## Results

### Performance on the original PPI networks

We first evaluated TOMOC on the three original, unperturbed PPI networks: Yeast-D1, Yeast-D2, and Collins. The predicted complexes were compared with Complex-D1, Complex-D2, and CYC2008, respectively, using a Jaccard matching threshold of *OS = 0.20*. Each experiment was repeated over 10 independent runs, and the solution with the highest F1-score was selected from the final nondominated archive in each run.

Table 1 summarizes the performance of TOMOC in terms of Precision, Recall, F1-score, and the number of predicted complexes. TOMOC achieved its highest mean F1-score on Yeast-D1 (*0.885 ± 0.014*), followed by Collins (*0.869 ± 0.008*) and Yeast-D2 (*0.843 ± 0.011*).

On Yeast-D1, TOMOC achieved a Precision of *0.875 ± 0.014* and a Recall of *0.896 ± 0.021*. On Yeast-D2, the method obtained its highest Recall, reaching *0.905 ± 0.022*, while maintaining a Precision of *0.788 ± 0.011*. On Collins, Precision and Recall were both approximately 0.869, resulting in an F1-score of *0.869 ± 0.008*.

The mean number of predicted complexes ranged from *153.0 ± 6.0* on Yeast-D1 to *307.8 ± 12.9* on Collins. The corresponding prediction-to-reference ratios ranged from 1.18 to 1.96, providing additional context regarding the number of predicted complexes relative to the corresponding reference complex sets.

**Table 1:** Performance of TOMOC on the original, unperturbed PPI networks using a Jaccard matching threshold of *OS = 0.20*. Results are reported as mean ± standard deviation across 10 independent runs

Dataset	#Reference	Precision	Recall	F1-score	#Predicted	Pred./Ref. ratio
Yeast-D1	78	*0.875 ± 0.014*	*0.896 ± 0.021*	*0.885 ± 0.014*	*153.0 ± 6.0*	*1.96 ± 0.08*
Yeast-D2	150	*0.788 ± 0.011*	*0.905 ± 0.022*	*0.843 ± 0.011*	*208.4 ± 11.4*	*1.39 ± 0.08*
Collins	261	*0.869 ± 0.016*	*0.869 ± 0.016*	*0.869 ± 0.008*	*307.8 ± 12.9*	*1.18 ± 0.05*

The coefficient of variation of the F1-score remained below 2% across all three datasets, indicating limited run-to-run variability. Detailed run-to-run stability statistics are provided in Additional file 2, Section S2.5 and Table S2.5.

### Comparison with representative overlapping protein-complex detection methods

To directly evaluate TOMOC against methods specifically designed for overlapping protein-complex detection, we conducted an additional comparison on the Gavin, Collins, and Krogan PPI networks. All three networks were evaluated against the CYC2008 reference complex set using the overlap-score criterion with a matching threshold of *OS = 0.20*.

The comparison includes ClusterONE [15], PEWCC [16], EGCPI [17], GMFTP [18], LADOC [19], CACO [20], AdaPPI [21], and FMvPCI [22].

The results of the competing methods were obtained from the published comparative evaluation reported by FMvPCI [22], which used the same PPI networks, CYC2008 reference complex set, and matching threshold. The competing methods were not rerun in our experimental environment. The TOMOC results correspond to the mean performance of the best-F1 solution selected from the final nondominated archive in each of 10 independent runs. Table 2 reports Precision, Recall, and F1-score.

**Table 2:** Complex-level comparison of TOMOC with representative overlapping protein-complex detection methods on the Gavin, Collins, and Krogan PPI networks. Evaluation was performed against CYC2008 using an overlap-score matching threshold of *OS = 0.20*. Results for the competing methods were obtained from a published comparative evaluation, whereas TOMOC values represent means across 10 independent runs.

Method	Gavin			Collins			Krogan		
	Precision	Recall	F1-score	Precision	Recall	F1-score	Precision	Recall	F1-score
ClusterONE	0.63	0.28	0.39	0.62	0.35	0.45	0.60	0.32	0.42
PEWCC	0.58	0.31	0.40	0.74	0.32	0.45	0.62	0.33	0.43
EGCPI	0.73	0.28	0.40	0.78	0.36	0.49	0.62	0.33	0.43
GMFTP	0.77	0.31	0.44	0.73	0.35	0.48	0.51	0.37	0.43
LADOC	0.49	0.37	0.42	0.65	0.41	0.51	0.45	0.43	0.44
CACO	**0**.**84**	0.42	0.56	**0**.**92**	0.40	0.56	**0**.**83**	0.28	0.42
AdaPPI	0.48	0.53	0.50	0.82	0.35	0.49	0.73	0.29	0.42
FMvPCI	0.61	0.58	**0**.**59**	0.72	0.53	0.61	0.42	**0**.**54**	**0**.**47**
**TOMOC**	0.540	**0**.**629**	0.580	0.686	**0**.**806**	**0**.**741**	0.400	0.512	0.449

Bold values denote the highest result for each metric and network

On the Gavin network, TOMOC achieved the highest Recall among all compared methods, reaching 0.629. Its F1-score of 0.580 was the second highest, closely approaching the value of 0.590 reported for FMvPCI. Although CACO achieved the highest Precision, its lower Recall resulted in a slightly lower F1-score. These results indicate that TOMOC recovered a comparatively large proportion of the CYC2008 reference complexes while maintaining competitive overall performance.

On the Collins network, TOMOC achieved the highest Recall and F1-score, reaching 0.806 and 0.741, respectively. The next-highest F1-score was 0.610, reported for FMvPCI. Although CACO and AdaPPI achieved higher Precision, their lower Recall values resulted in lower F1-scores. TOMOC therefore provided the strongest balance between Precision and Recall among the compared methods on the Collins network.

On the Krogan network, FMvPCI achieved the highest Recall and F1-score, with values of 0.540 and 0.470, respectively. TOMOC obtained the second-highest Recall and F1-score, reaching 0.512 and 0.449. Although its Precision was lower than that of several competing methods, TOMOC remained competitive in terms of reference-complex recovery and overall F1 performance.

Additional comparisons with cut-based genetic algorithms, canonical single-objective evolutionary algorithms, heuristic-enhanced variants, and multi-objective evolutionary algorithms on the Collins PPI network are provided in Additional file 2, Sections S2.1-S2.4 (Tables S2.1-S2.4).

### Comparison with evolutionary baselines

To assess the contribution of the multi-objective formulation adopted by TOMOC, we compared the proposed framework with three groups of evolutionary baselines: canonical single-objective evolutionary algorithms, heuristic-enhanced single-objective evolutionary algorithms, and multi-objective evolutionary algorithms.

The canonical single-objective methods optimize one of six structural criteria: community score (EA-CS), expansion (EA-EX), ratio cut (EA-RC), normalized cut (EA-NC), internal density (EA-ID), or modularity (EA-Q), following the evolutionary formulations described by Pizzuti et al. [27]. Their heuristic-enhanced variants, denoted by the subscript *mu*, incorporate a topology-guided mutation mechanism while retaining a single-objective formulation  [41]. The multi-objective baselines include MOCD$_1$, proposed by Bandyopadhyay et al.  [28], and MOCD$_2$, proposed by Attea et al. [29].

All methods were assessed on the Yeast-D1 and Yeast-D2 datasets using the same complex-level evaluation protocol and a Jaccard matching threshold of *OS = 0.20*. For TOMOC, the reported results correspond to the mean performance of the best-F1 solution selected from the final nondominated archive in each of 10 independent runs.

Table 3 consolidates the comparisons that were previously presented separately for the three groups of evolutionary algorithms.

**Table 3:** Complex-level performance comparison between TOMOC and evolutionary baseline methods on the Yeast-D1 and Yeast-D2 datasets using a Jaccard matching threshold of *OS = 0.20*. The baseline methods are ordered according to their evolutionary families. TOMOC results are reported as means across 10 independent runs, with the standard deviation provided for the F1-score.

Algorithm	Yeast-D1			Yeast-D2		
	Precision	Recall	F1-score	Precision	Recall	F1-score
EA-CS	0.6925	0.8359	0.7571	0.4337	0.7247	0.5424
EA-EX	0.6810	0.7603	0.7180	0.4404	0.6933	0.5381
EA-RC	0.7096	0.7000	0.7046	0.4562	0.6533	0.5372
EA-NC	0.7081	0.6782	0.6924	0.4624	0.6293	0.5330
EA-ID	0.6474	0.7115	0.6776	0.4186	0.6600	0.5120
EA-Q	0.6910	0.7051	0.6977	0.4504	0.6760	0.5405
EA-CS$_{mu}$	0.6990	0.8628	0.7722	0.4339	0.7507	0.5496
EA-EX$_{mu}$	0.6550	0.8090	0.7237	0.4243	0.7033	0.5290
EA-RC$_{mu}$	0.7110	0.6167	0.6601	0.4604	0.5940	0.5183
EA-NC$_{mu}$	0.7280	0.6385	0.6800	0.4764	0.6213	0.5391
EA-ID$_{mu}$	0.6299	0.7321	0.6767	0.4194	0.6667	0.5148
EA-Q$_{mu}$	0.7100	0.6205	0.6620	0.4674	0.5967	0.5238
$MOCD_1$	0.7327	0.8949	0.7745	0.4766	0.7893	0.5823
$MOCD_2$	0.7394	0.8923	0.7774	0.4927	0.7667	0.5714
**TOMOC**	**0**.**8752**	**0**.**8962**	$\mathbf {0.8854 \pm 0.0143}$	**0**.**7884**	**0**.**9053**	$\mathbf {0.8427 \pm 0.0114}$

Bold values indicate the highest result for each metric and dataset

On Yeast-D1, TOMOC achieved the highest values for all three evaluation metrics, with a Precision of 0.8752, a Recall of 0.8962, and an F1-score of *0.8854 ± 0.0143*. Among the baseline methods, MOCD$_2$ obtained the highest F1-score of 0.7774, followed by MOCD$_1$ with 0.7745. TOMOC therefore maintained higher Precision and Recall, resulting in the strongest overall F1-score.

On Yeast-D2, TOMOC also achieved the highest values for all three evaluation metrics, with a Precision of 0.7884, a Recall of 0.9053, and an F1-score of *0.8427 ± 0.0114*. The strongest baseline F1-score was 0.5823, obtained by MOCD$_1$. The larger performance difference on Yeast-D2 was primarily associated with the substantially higher Precision maintained by TOMOC while preserving high Recall.

Among the canonical single-objective methods, EA-CS produced the highest F1-score on both datasets, reaching 0.7571 on Yeast-D1 and 0.5424 on Yeast-D2. Its heuristic-enhanced variant, EA-CS$_{mu}$, increased these values to 0.7722 and 0.5496, respectively. However, both variants remained below TOMOC, particularly on Yeast-D2.

The multi-objective baselines generally achieved stronger performance than the single-objective methods on Yeast-D1, but their Precision decreased considerably on Yeast-D2. In contrast, TOMOC maintained a more balanced Precision-Recall profile across both datasets. These results support the use of complementary structural objectives for balancing internal cohesion and boundary separation during overlapping protein-complex detection.

### Statistical significance analysis

To assess whether the performance differences achieved by TOMOC are statistically significant, we conducted a non-parametric Wilcoxon signed-rank test using the run-level F1-scores obtained from 10 runs. TOMOC was compared with the canonical and heuristic-guided evolutionary baselines MOCD$_1$, MOCD$_2$, EA-Q, and EA-Q$_{mu}$ across all evaluated datasets.

For consistency with the main performance tables, the run-level F1-scores used in the statistical analysis were obtained using the same validation procedures employed to generate the corresponding reported results. For each run, the best F1-score was retained using the matching threshold *OS=0.20*. The resulting run-level scores were then compared using the two-sided Wilcoxon signed-rank test.

As summarized in Table 4, all pairwise comparisons produced statistically significant differences at *α =0.05*. Across the four datasets and four baseline methods, all comparisons yielded *p=0.0019531*, indicating consistent differences between TOMOC and the evaluated evolutionary baselines across the analyzed runs.

**Table 4:** Wilcoxon signed-rank test comparing TOMOC with evolutionary baselines across the evaluated datasets. Results are based on run-level F1-scores from 10 runs and are reported as mean ± standard deviation. Statistical significance was assessed at *α =0.05*

Dataset	Baseline	TOMOC	Baseline	p-value
Yeast-D1	MOCD$_1$	0.8854 ± 0.0143	0.7745 ± 0.0111	0.0019531
Yeast-D1	MOCD$_2$	0.8854 ± 0.0143	0.7774 ± 0.0140	0.0019531
Yeast-D1	EA-Q	0.8854 ± 0.0143	0.6977 ± 0.0213	0.0019531
Yeast-D1	EA-Q$_{mu}$	0.8854 ± 0.0143	0.6620 ± 0.0166	0.0019531
Yeast-D2	MOCD$_1$	0.8427 ± 0.0114	0.5823 ± 0.0119	0.0019531
Yeast-D2	MOCD$_2$	0.8427 ± 0.0114	0.5714 ± 0.0137	0.0019531
Yeast-D2	EA-Q	0.8427 ± 0.0114	0.5405 ± 0.0220	0.0019531
Yeast-D2	EA-Q$_{mu}$	0.8427 ± 0.0114	0.5238 ± 0.0148	0.0019531
Collins-CYC2008	MOCD$_1$	0.8690 ± 0.0080	0.7153 ± 0.0069	0.0019531
Collins-CYC2008	MOCD$_2$	0.8690 ± 0.0080	0.7165 ± 0.0043	0.0019531
Collins-CYC2008	EA-Q	0.8690 ± 0.0080	0.6777 ± 0.0141	0.0019531
Collins-CYC2008	EA-Q$_{mu}$	0.8690 ± 0.0080	0.6486 ± 0.0141	0.0019531
Collins-MIPS	MOCD$_1$	0.8733 ± 0.0053	0.5655 ± 0.0078	0.0019531
Collins-MIPS	MOCD$_2$	0.8733 ± 0.0053	0.5622 ± 0.0084	0.0019531
Collins-MIPS	EA-Q	0.8733 ± 0.0053	0.5263 ± 0.0092	0.0019531
Collins-MIPS	EA-Q$_{mu}$	0.8733 ± 0.0053	0.5246 ± 0.0136	0.0019531

On Yeast-D1, TOMOC achieved significantly higher run-level F1-scores than MOCD$_1$, MOCD$_2$, EA-Q, and EA-Q$_{mu}$. The performance differences were also statistically significant on Yeast-D2, where TOMOC maintained higher mean F1-scores than all four evolutionary baselines.

For the Collins network, statistically significant differences were observed using both the CYC2008 and MIPS reference-complex sets. TOMOC achieved higher mean F1-scores than the multi-objective baselines MOCD$_1$ and MOCD$_2$, as well as the single-objective EA-Q and EA-Q$_{mu}$ methods.

### Parameter sensitivity analysis

To assess the sensitivity of the reported performance to the complex-matching threshold, we conducted an analysis using three commonly adopted Jaccard threshold values, $OS \in \{0.1,0.2,0.3\}$. This threshold controls the strictness of the matching criterion between predicted and reference complexes and is applied only during post-hoc performance evaluation; it does not affect the TOMOC optimization process. For each threshold, the solution with the highest F1-score was selected from the final nondominated archive in each run, and the reported values correspond to the mean and standard deviation across 10 independent runs.

The results, summarized in Table 5, reveal a consistent and interpretable trend across all evaluated settings. Under the more permissive setting (*OS=0.1*), TOMOC achieves the highest F1-scores, reaching *0.9836 ± 0.0055* on Yeast-D1, *0.9522 ± 0.0088* on Yeast-D2, *0.9422 ± 0.0060* on Collins-CYC2008, and *0.9418 ± 0.0076* on Collins-MIPS. This behavior is expected because a lower threshold allows a larger proportion of predicted complexes to be matched with the reference complexes, thereby increasing both Recall and the resulting F1-score.

When the threshold is increased to the primary evaluation setting used in this study (*OS=0.2*), the F1-scores decrease to *0.8854 ± 0.0143*, *0.8427 ± 0.0114*, *0.8690 ± 0.0080*, and *0.8733 ± 0.0053* for Yeast-D1, Yeast-D2, Collins-CYC2008, and Collins-MIPS, respectively. These values correspond to the Jaccard matching threshold used in the main benchmark experiments.

Under the stricter setting (*OS=0.3*), the F1-scores decline further, reaching *0.7907 ± 0.0157* on Yeast-D1, *0.7052 ± 0.0149* on Yeast-D2, *0.7763 ± 0.0117* on Collins-CYC2008, and *0.7702 ± 0.0144* on Collins-MIPS. This reduction is primarily caused by the stricter matching requirement, which makes partial agreement between predicted and reference complexes less likely to be accepted.

Overall, the decline in Precision, Recall, and F1-score was gradual as the Jaccard matching threshold became stricter, and the same general pattern was observed across all four evaluation settings. These results demonstrate the expected influence of the external matching criterion on the reported benchmark performance.

**Table 5:** Sensitivity of TOMOC performance to the Jaccard complex-matching threshold *OS* across the evaluated settings. Results are reported as mean ± standard deviation across 10 independent runs

Dataset	*OS*	F1-score	Precision	Recall
Yeast-D1	0.1	0.9836 ± 0.0055	0.9815 ± 0.0091	0.9859 ± 0.0127
Yeast-D1	0.2	0.8854 ± 0.0143	0.8752 ± 0.0139	0.8962 ± 0.0205
Yeast-D1	0.3	0.7907 ± 0.0157	0.7593 ± 0.0139	0.8256 ± 0.0321
Yeast-D2	0.1	0.9522 ± 0.0088	0.9209 ± 0.0153	0.9860 ± 0.0073
Yeast-D2	0.2	0.8427 ± 0.0114	0.7884 ± 0.0111	0.9053 ± 0.0217
Yeast-D2	0.3	0.7052 ± 0.0149	0.6388 ± 0.0208	0.7880 ± 0.0255
Collins-CYC2008	0.1	0.9422 ± 0.0060	0.9415 ± 0.0096	0.9429 ± 0.0106
Collins-CYC2008	0.2	0.8690 ± 0.0080	0.8694 ± 0.0157	0.8690 ± 0.0161
Collins-CYC2008	0.3	0.7763 ± 0.0117	0.7822 ± 0.0143	0.7709 ± 0.0228
Collins-MIPS	0.1	0.9418 ± 0.0076	0.9285 ± 0.0121	0.9557 ± 0.0074
Collins-MIPS	0.2	0.8733 ± 0.0053	0.8549 ± 0.0112	0.8927 ± 0.0083
Collins-MIPS	0.3	0.7702 ± 0.0144	0.7680 ± 0.0163	0.7726 ± 0.0176

An exploratory analysis was conducted to examine the relationships between the two optimized topological objectives and external F1-score across the pooled nondominated solutions. The results showed a consistent negative association between Obj1 and Obj2 across the evaluated datasets, while the strength of their associations with external F1-score varied by dataset. The complete correlation analysis and Pareto-front visualizations are provided in Additional file 2, Section S2.6.

### Robustness under network perturbations

To evaluate the robustness of TOMOC to uncertainty in PPI data, we examined its performance under controlled interaction deletion and insertion. The analysis was conducted across four evaluation settings: Yeast-D1, Yeast-D2, Collins-CYC2008, and Collins-MIPS. The latter two settings use the same Collins PPI network but different reference complex sets.

Interactions were deleted or inserted at perturbation levels ranging from 10% to 50%. For both perturbation types, three interaction-selection strategies were considered: random selection, prioritization of interactions associated with high-degree proteins, and prioritization of interactions associated with low-degree proteins. Performance was evaluated using the Jaccard-based protocol with *OS=0.20*. For each perturbation setting, the reported F1-score represents the mean performance of the representative solutions selected from the final nondominated archives across 10 independent runs.

**Fig. 3:**
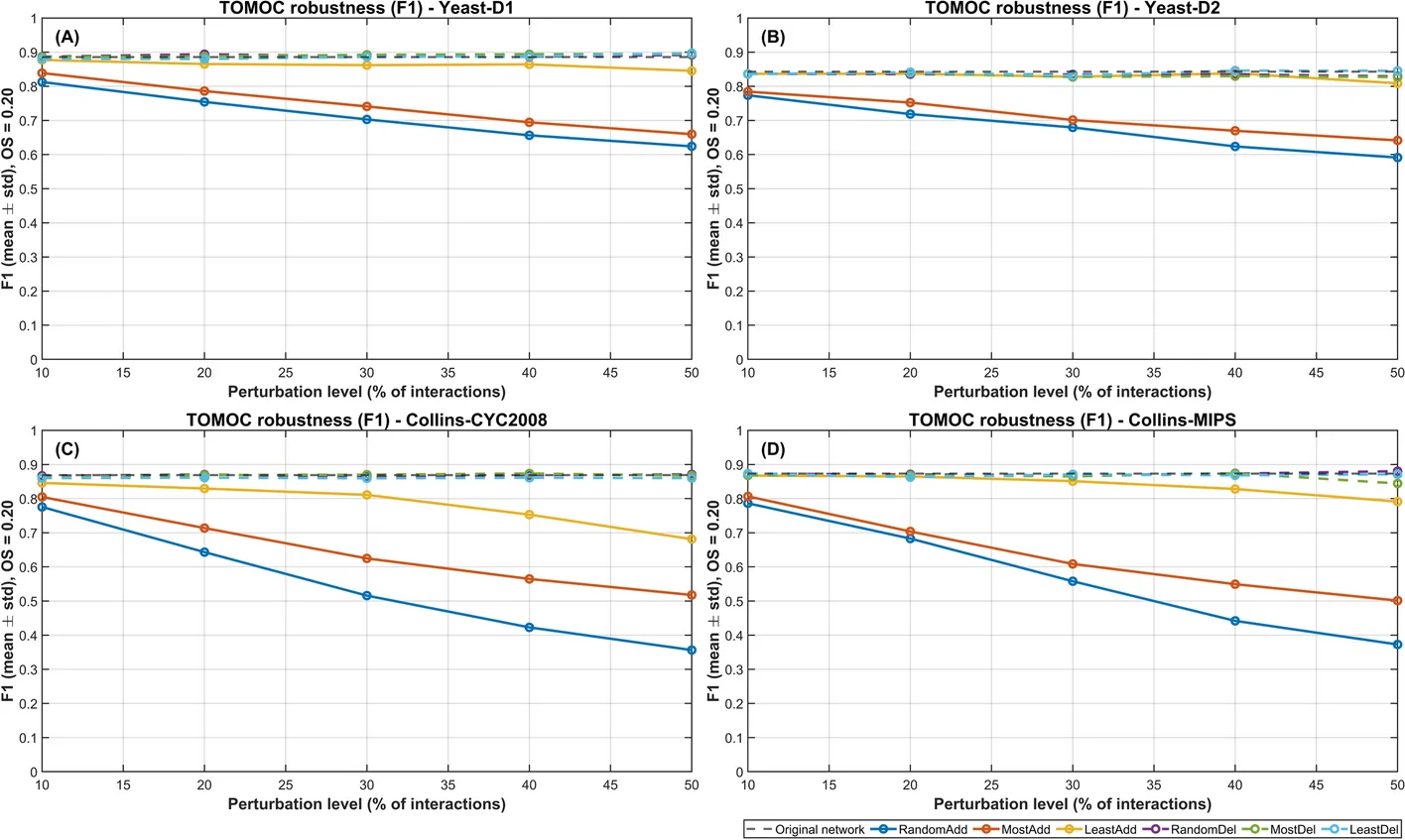
F1-score performance of TOMOC under multi-level interaction deletion and insertion. Panels correspond to **A** Yeast-D1, **B** Yeast-D2, **C** Collins-CYC2008, and **D** Collins-MIPS. Perturbation levels range from 10% to 50%, and the curves represent the mean F1-score across 10 independent runs using a Jaccard matching threshold of *OS = 0.20*. The results show comparatively limited degradation under interaction deletion and a more pronounced decline when spurious interactions are added

Figure 3 shows the evolution of the F1-score as the perturbation level increases. Across the four evaluation settings, TOMOC generally exhibited a gradual rather than abrupt reduction in performance. Deletion-based perturbations produced comparatively limited changes, particularly at lower and moderate perturbation levels. This pattern suggests that the method can retain useful structural information when a proportion of the original interactions is missing.

In contrast, the addition of spurious interactions produced a more noticeable decline in F1-score. The effect became progressively stronger as the proportion of inserted interactions increased. Added interactions may connect otherwise distinct network regions, thereby reducing the separation between candidate complexes and modifying local connectivity patterns used by the two structural objectives.

The magnitude of performance degradation varied across the evaluation settings and perturbation strategies. Nevertheless, the overall trends were consistent: deletion-based noise was generally less disruptive than insertion-based noise, while higher perturbation levels resulted in greater performance loss. These findings indicate that TOMOC retains a degree of stability under incomplete and noisy PPI networks, although its performance remains sensitive to substantial levels of false-positive interactions.

Additional summaries of the relative F1-score degradation, including perturbation-specific curves, heatmaps, and average degradation rates, are provided in Additional file 2, Section S2.7 (Figs. S2.4–S2.6).

### Independent go-based biological validation

To assess whether the protein complexes detected by TOMOC exhibit functional coherence, we performed an independent post-hoc validation using gene ontology (GO) annotations. GO information was not used during solution encoding, objective evaluation, evolutionary optimization, mutation, decoding, Pareto archive construction, or final-solution selection. It was used exclusively after complex detection as an external source of biological evidence.

The biological evaluation comprised two complementary quantitative analyses and an illustrative case-study assessment. First, GO semantic coherence was used to quantify the functional similarity among the proteins assigned to each predicted complex. Second, GO enrichment analysis was performed separately for the Biological Process (BP), Molecular Function (MF), and Cellular Component (CC) domains. Finally, representative TOMOC predictions were examined in relation to established yeast protein complexes.

#### GO semantic coherence

For each predicted complex containing at least two GO-annotated proteins, we calculated the mean pairwise semantic similarity among its annotated members. The observed coherence was compared with that of 1000 size-matched random protein sets. Empirical *p*-values were adjusted using the Benjamini-Hochberg procedure, and a predicted complex was considered significantly coherent when *q < 0.05* and its observed similarity exceeded the corresponding random expectation.

Table 6 summarizes the results across 10 independent runs. Annotation coverage exceeded 85% on all three networks. The mean GO coherence of the TOMOC complexes was consistently higher than the size-matched random expectation.

The largest difference was observed on Collins, where the mean semantic coherence reached *0.7299 ± 0.0045*, compared with *0.4644 ± 0.0001* for the random controls. On Gavin and Krogan, the corresponding differences were *0.1681 ± 0.0097* and *0.1346 ± 0.0077*, respectively.

Moreover, $88.87\% \pm 1.38\%$ of the evaluated Collins complexes showed significant GO coherence. The corresponding proportions were $70.51\% \pm 2.44\%$ on Gavin and $54.48\% \pm 3.56\%$ on Krogan. These results indicate that the complexes detected by TOMOC frequently group proteins with related functional annotations, despite the absence of GO information from the optimization process.

**Table 6:** Post-hoc GO semantic-coherence analysis of TOMOC complexes. Annotation coverage denotes the proportion of proteins in the evaluated complexes with available GO annotations. Random expectation was estimated using 1000 size-matched random protein sets. Significant complexes satisfied *q < 0.05* and had observed coherence greater than the corresponding random expectation. Values are reported as mean ± standard deviation across 10 independent runs

Dataset	Annotation coverage	TOMOC coherence	Random expectation	Difference	Significant complexes
Collins	*0.8597 ± 0.0056*	*0.7299 ± 0.0045*	*0.4644 ± 0.0001*	*0.2655 ± 0.0044*	$88.87\% \pm 1.38\%$
Gavin	*0.8755 ± 0.0088*	*0.6276 ± 0.0097*	*0.4596 ± 0.0001*	*0.1681 ± 0.0097*	$70.51\% \pm 2.44\%$
Krogan	*0.8751 ± 0.0079*	*0.5703 ± 0.0076*	*0.4357 ± 0.0001*	*0.1346 ± 0.0077*	$54.48\% \pm 3.56\%$

#### GO enrichment analysis

We next evaluated whether the predicted complexes were significantly enriched for specific GO terms. Enrichment was assessed independently within the BP, MF, and CC domains using the upper-tail hypergeometric test. The resulting *p*-values were corrected separately for each predicted complex and GO domain using the Benjamini-Hochberg procedure.

A predicted complex was considered enriched in a GO domain when at least one term satisfied the false-discovery-rate threshold of *q < 0.05*. Table 7 reports the proportion of predicted complexes with significant enrichment in each GO domain and in at least one domain.

On Collins, $95.31\% \pm 1.07\%$ of the evaluated complexes were enriched in at least one GO domain. High enrichment rates were also observed on Gavin and Krogan, reaching $88.65\% \pm 1.68\%$ and $76.94\% \pm 1.39\%$, respectively.

Across all three networks, BP and CC enrichment occurred more frequently than MF enrichment. This pattern was particularly evident on Collins, where $92.32\% \pm 1.49\%$ of the complexes were enriched for BP terms and $89.96\% \pm 1.03\%$ were enriched for CC terms. Collectively, these results provide complementary evidence that a substantial proportion of the topologically detected complexes correspond to functionally associated protein groups.

**Table 7:** Percentage of TOMOC complexes with significant GO enrichment in the Biological Process (BP), Molecular Function (MF), and Cellular Component (CC) domains. “Any domain” denotes enrichment in at least one of the three GO domains. Values are reported as mean ± standard deviation across 10 independent runs

Dataset	BP	MF	CC	Any domain
Collins	$92.32\% \pm 1.49\%$	$69.90\% \pm 1.86\%$	$89.96\% \pm 1.03\%$	$95.31\% \pm 1.07\%$
Gavin	$81.80\% \pm 1.70\%$	$60.51\% \pm 3.35\%$	$82.68\% \pm 1.36\%$	$88.65\% \pm 1.68\%$
Krogan	$69.33\% \pm 2.48\%$	$48.33\% \pm 1.98\%$	$70.29\% \pm 1.39\%$	$76.94\% \pm 1.39\%$

#### Representative biological case studies

To illustrate the biological relevance of individual TOMOC predictions, Table 8 presents five representative cases associated with well-characterized yeast protein complexes. The selected cases include the mitochondrial ribosome, proteasome, RNA polymerase, INO80, and Mediator complexes.

The Collins mitochondrial-ribosome prediction showed complete reference coverage and an overlap score of 0.9310. The Gavin proteasome prediction also covered all proteins in the corresponding reference complex. On Krogan, the RNA polymerase, INO80, and Mediator predictions recovered substantial proportions of their reference complexes and exhibited highly significant GO enrichment.

These examples illustrate that the topology-only optimization can identify protein groups corresponding to established cellular assemblies. The representative matches, adjusted significance values, overlap scores, and reference coverage are summarized in Table 8.

**Table 8:** Representative TOMOC predictions associated with established yeast protein complexes. The adjusted *q*-value corresponds to the representative enriched GO term reported for each prediction. OS denotes the overlap score between the predicted and reference complexes, and reference coverage denotes the proportion of reference-complex proteins recovered by the TOMOC prediction

Dataset	Representative complex	Representative enriched GO term	Adjusted *q*-value	OS	Reference coverage (%)
Collins	Mitochondrial ribosome	Organellar large ribosomal subunit (GO:0000315; CC)	$2.9588 \times 10^{-60}$	0.9310	100
Gaviin	Proteasome	Proteasome complex (GO:0000502; CC)	$2.4329 \times 10^{-39}$	0.4000	100
Krogan	RNA polymerase	5$^{\prime }$–3$^{\prime }$ RNA polymerase activity (GO:0034062; MF)	$5.6670 \times 10^{-41}$	0.3977	76.47
Krogan	INO80	INO80-type complex (GO:0097346; CC)	$1.9422 \times 10^{-35}$	0.4332	81.82
Krogan	Mediator	Core mediator complex (GO:0070847; CC)	$7.9160 \times 10^{-37}$	0.5635	72.00

The enriched GO annotations were consistent with the established biological roles of the corresponding protein assemblies. The Collins prediction matched the mitochondrial ribosome with complete reference coverage and showed strong enrichment for the organellar large ribosomal subunit, supporting its role in mitochondrial protein synthesis. The Gavin prediction recovered the complete reference proteasome and was enriched for the proteasome complex, indicating coherent identification of proteins involved in regulated protein degradation. For the Krogan network, the RNA-polymerase prediction was associated with RNA-polymerase activity, whereas the INO80 and Mediator predictions were enriched for the INO80-type and core Mediator complexes, respectively. These annotations are consistent with their established roles in transcription, chromatin remodeling, and transcriptional regulation.

### Computational complexity, runtime, and scalability analysis

In addition to predictive performance, it is essential to assess the computational feasibility of the proposed TOMOC framework, particularly given its edge-based encoding scheme and multi-objective evolutionary optimization strategy. Unlike node-based representations, TOMOC operates in a search space proportional to the number of interactions (|*E*|), which is typically significantly larger than the number of proteins (|*V*|) in PPI networks. This characteristic raises important questions regarding computational complexity and scalability when applied to large and dense biological networks.

To address this, we conducted an empirical runtime analysis across four evaluation settings: Yeast-D1, Yeast-D2, Collins-CYC2008, and Collins-MIPS. The latter two settings use the same Collins PPI network but different reference complex sets. All experiments were executed under identical parameter settings, with a population size of 100 individuals and 100 evolutionary generations. This corresponds to 10,000 solution evaluations per run and 100,000 solution evaluations across the 10 independent runs conducted for each evaluation setting.

Table 9 summarizes the total runtime and average runtime per run for each dataset. The results show a clear and consistent increase in computational cost as the network size and interaction density grow. The average runtime per run increases from 275.57 seconds on Yeast-D1 to 655.66 seconds on Collins-MIPS, reflecting the additional cost associated with evaluating larger and more complex interaction structures.

**Table 9:** Runtime analysis of TOMOC across benchmark PPI networks. Each experiment consists of 10 independent runs under identical parameter settings

Dataset	Total runtime (s)	Avg runtime/run (s)	Evaluations
Yeast-D1	2755.7	275.57	$10^5$
Yeast-D2	4664.3	466.43	$10^5$
Collins-CYC2008	6469.4	646.94	$10^5$
Collins-MIPS	6556.6	655.66	$10^5$

From a computational perspective, the dominant cost in TOMOC arises from two main components: (i) decoding edge-based chromosomes into overlapping protein complexes, and (ii) evaluating structural objectives, including conductance and triangle-density loss. The decoding process involves mapping edge assignments to node-level cluster memberships, which requires traversing interaction structures and resolving overlaps. The objective evaluation further requires computing boundary cuts and local triangular structures, both of which depend on the connectivity patterns of the network.

Consequently, the overall computational complexity per generation can be approximated as *O(P · (|E| + T))*, where *P* is the population size and *T* denotes the number of triangle-related operations required for cohesion evaluation. Across *G* generations, the total complexity becomes $O(G \cdot P \cdot (|E| + T))$. Although this formulation reflects the higher computational demand associated with edge-based representations, it also highlights that the complexity scales primarily with the number of interactions rather than exhibiting exponential growth.

To further examine scalability, we analyze the relationship between runtime and network size. As observed in Table 9, the runtime increases progressively from yeast datasets to the larger Collins networks, consistent with the growth in interaction density and network size. Importantly, this increase remains controlled and does not exhibit exponential behavior. Instead, the observed trend suggests a near-linear to sub-quadratic scaling pattern with respect to |*E*|.

This controlled scaling can be attributed to the decomposition-based optimization strategy employed by MOEA/D. By decomposing the multi-objective problem into a set of scalar subproblems and restricting interactions within local neighborhoods, the algorithm effectively distributes the search process and limits computational overhead. Furthermore, the use of a fixed evaluation budget ($10^5$ solution evaluations across 10 independent runs) ensures that scalability is achieved through efficient optimization rather than reduced computational effort.

## Discussion

### Main findings

This study introduced TOMOC, a topology-driven multi-objective evolutionary framework for overlapping protein-complex detection in PPI networks. The framework combines an edge-based representation with two complementary structural objectives: average conductance and triangle-density loss. Together, these components allow TOMOC to search for overlapping modules that exhibit both internal cohesiveness and separation from the surrounding network.

The results on the original PPI networks showed that TOMOC maintained consistently high F1-scores across Yeast-D1, Yeast-D2, and Collins (Table 1). The relatively low run-to-run variability suggests that the evolutionary search produced stable outcomes under the adopted experimental settings. The balance between Precision and Recall varied across the networks, but TOMOC maintained comparatively strong overall performance in all three cases.

The comparison with representative overlapping protein-complex detection methods further clarified the behavior of TOMOC (Table 2). TOMOC achieved the highest Recall on Gavin and Collins and the highest F1-score on Collins. It also obtained the second-highest Recall and F1-score on Krogan. These results suggest that the method is particularly effective at recovering reference complexes, although its Precision on Gavin and Krogan was lower than that of several competing approaches. This pattern reflects a trade-off between recovering a larger proportion of the reference complexes and limiting unmatched predictions.

The evolutionary-baseline comparison provides additional evidence regarding the role of the multi-objective formulation (Table 3). TOMOC achieved the highest F1-score on both Yeast-D1 and Yeast-D2 and maintained a more balanced Precision-Recall profile than the evaluated single-objective and multi-objective baselines. Optimizing conductance and triangle-density loss simultaneously therefore appears to provide a useful compromise between external separation and internal cohesion. Neither structural property alone is expected to characterize all biologically relevant complex topologies, whereas the nondominated archive preserves alternative trade-off solutions throughout the evolutionary process.

The edge-based representation is also central to TOMOC’s ability to detect overlapping structures. Because memberships are derived from interaction patterns rather than assigning each protein to a single exclusive cluster, the same protein can occur in multiple decoded complexes. This representation is consistent with the multifunctional role of proteins that participate in different cellular assemblies. Overlapping membership therefore emerges from the encoded interaction structure rather than being introduced only through a post-processing step.

The perturbation experiments showed that TOMOC retained a degree of stability under both missing and spurious interactions (Fig. 3). Deletion-based perturbations generally caused smaller changes in F1-score, whereas interaction insertion produced a more pronounced decline as the perturbation level increased. Missing interactions may weaken local structures without necessarily introducing connections between unrelated modules. In contrast, spurious interactions can connect otherwise distinct network regions and alter both boundary separation and local clustering patterns. The results should therefore be interpreted as evidence of gradual performance degradation rather than complete invariance to network noise.

The post-hoc GO analyses provide independent biological support for the detected complexes. TOMOC complexes exhibited higher semantic coherence than size-matched random protein sets across Gavin, Collins, and Krogan (Table 6). A substantial proportion of the complexes were also significantly enriched in at least one GO domain (Table 7). The representative case studies further showed correspondence with established assemblies such as the mitochondrial ribosome, proteasome, RNA polymerase, INO80, and Mediator complexes (Table 8). Importantly, GO annotations were not used during encoding, optimization, objective evaluation, decoding, Pareto-archive generation, or solution selection. The GO results therefore represent independent post-hoc evidence rather than information incorporated into the search process.

### Limitations and future directions

Several limitations should be considered when interpreting the results. First, the experimental evaluation was restricted primarily to yeast PPI networks and yeast reference complex sets. These datasets are widely used and support comparison with many existing methods, but they do not fully represent the scale, sparsity, annotation incompleteness, or biological complexity of higher-organism interactomes. Evaluation on human, mouse, and other organism-specific PPI networks is therefore required before broader generalization.

Second, TOMOC relies exclusively on network topology during optimization. This design enables biological annotations to remain independent during post-hoc validation, but it also means that the search does not directly use functional, expression, localization, or temporal information. Integrating such evidence as additional objectives or carefully designed priors may improve functional specificity, particularly in networks containing substantial topological noise. However, any biologically informed extension should be evaluated separately from the topology-only formulation to preserve clarity regarding the source of performance improvements.

Third, the edge-based representation and repeated structural evaluation introduce computational overhead. Chromosome decoding, conductance calculation, and triangle-related evaluation become increasingly expensive as network size and density increase. The runtime results in Table 9 indicate that TOMOC is computationally feasible for the evaluated networks, but additional optimization may be needed for large-scale interactomes. Parallel objective evaluation, efficient triangle-counting procedures, incremental fitness updates, and sparse data structures represent promising directions for improving scalability.

Fourth, some comparative results were obtained from published evaluations rather than by rerunning all competing implementations within a unified software environment. Although the selected comparisons used the same networks, reference sets, and matching criteria, implementation details and preprocessing choices may still differ across studies. Future work could include a fully reproducible benchmark in which all available methods are executed under a common preprocessing and evaluation pipeline.

Finally, the current perturbation analysis examines controlled interaction addition and deletion. These experiments approximate common sources of uncertainty in PPI data, but they do not capture all forms of biological and experimental variability. Future studies could consider confidence-weighted interactions, condition-specific networks, dynamic or temporal PPIs, and network perturbations informed by experimental reliability scores.

## Conclusions

This work presented TOMOC, a topology-driven multi-objective evolutionary framework for detecting overlapping protein complexes in PPI networks. TOMOC formulates the problem using two complementary structural objectives: average conductance, which promotes separation from the surrounding network, and triangle-density loss, which evaluates internal topological cohesion. Its edge-centric representation enables proteins to participate in multiple predicted complexes without requiring a predefined number of complexes, explicit overlap constraints, or biological annotations during optimization.

The experimental results showed that TOMOC achieved consistently strong performance on the original Yeast-D1, Yeast-D2, and Collins networks. It also performed competitively against representative overlapping protein-complex detection methods on the Gavin, Collins, and Krogan networks and achieved higher F1-scores than the evaluated evolutionary baselines on Yeast-D1 and Yeast-D2. Under controlled interaction deletion and insertion, the method generally exhibited gradual performance degradation, with interaction insertion producing a greater effect than interaction deletion. Furthermore, the independent post-hoc GO analyses showed that many TOMOC complexes exhibited higher semantic coherence than size-matched random protein sets and significant enrichment in Biological Process, Molecular Function, or Cellular Component terms. Because GO information was not incorporated into the evolutionary process, these findings provide independent biological support for the topology-derived predictions.

Several directions remain for future investigation. Incorporating interaction confidence scores may improve performance on networks containing uncertain or probabilistic edges. Extending TOMOC to multi-layer, condition-specific, or temporal biological networks could provide additional structural evidence for complex detection. Computational scalability may also be improved through incremental triangle counting, sparse data structures, parallel objective evaluation, and distributed evolutionary search. Finally, evaluation on larger and more diverse organism-specific interactomes will be necessary to determine the broader applicability of the proposed framework.

## Additional file

Supplementary Material 1

Supplementary Material 2

Supplementary Material 3

Supplementary Material 4

## Data Availability

The datasets analyzed in this study are publicly available. The Yeast-D1 and Yeast-D2 protein–protein interaction (PPI) datasets were derived from previously published yeast interaction maps reported by Gavin et al. [33] and further filtered following the procedure described by Zaki et al. [34]. The Collins PPI dataset was obtained from curated yeast interaction data available through the BioGRID database [35], which is publicly accessible at https://thebiogrid.org/. Reference protein complex datasets used for evaluation include the MIPS (Munich Information Center for Protein Sequences) complex catalog [36] and the CYC2008 curated yeast protein complex catalog [37]. The CYC2008 dataset is publicly available at http://wodaklab.org/cyc2008/. The MATLAB implementation of TOMOC, together with the processed datasets used in this study, is publicly available at https://github.com/mstmar92/TOMOC. All datasets used in this study originate from the cited publications and publicly accessible repositories.
