# Supplementary material for: TOMOC: a topology-driven multi-objective evolutionary framework for robust overlapping protein complex detection in noisy PPI networks
Source: BMC Bioinformatics. 2026 Aug 18;27:189. doi: 10.1186/s12859-026-06603-z (PMC13488024; doi:10.1186/s12859-026-06603-z)
Supplement: Supplementary file 1 [file 12859_2026_6603_MOESM1_ESM.pdf]

Additional file 1: Supplementary material

Algorithm S1: Decoding of an Edge-Based Individual

Input: Graph $G = (V, E)$, chromosome I
Output: Overlapping node complexes $\mathcal{C}$
$VisitedEdges \leftarrow \emptyset$
$EdgeComplexes \leftarrow \emptyset$
for each edge $e_i \in E$ **do**
- **if** $e_i \notin VisitedEdges$ **then**
  - $CplxE \leftarrow \emptyset$
  - $current \leftarrow e_i$
  - **while** $current \notin VisitedEdges$ **do**
    - add $current$ to $CplxE$
    - mark $current$ as visited
    - $current \leftarrow e_{I_{index(current)}}$
  - **end while**
  - add $CplxE$ to $EdgeComplexes$
- **end if**

end for
$\mathcal{C} \leftarrow \emptyset$
for each $CplxE \in EdgeComplexes$ **do**
- $CplxN \leftarrow \{u, v \mid (u, v) \in CplxE\}$
- add $CplxN$ to $\mathcal{C}$

end for
return $\mathcal{C}$

Algorithm S2: Structural Overlap Refinement (SOR)

Input: Decoded overlapping complexes $\mathcal{C} = \{C_1, \ldots, C_K\}$, adjacency matrix A, mutation probability p_m, thresholds τ, γ
Output: Refined overlapping structure $\mathcal{C}$
for each complex $C_k \in \mathcal{C}$ **do**
  for each protein $P_i \in V$ **do**
    if $rand() \leq p_m$ **then**
      {Stage 1: Boundary-aware Repair}
      if $P_i \in C_k$ **then**
        Compute $d_{\text{in}}(P_i, C_k) = \sum_{P_j \in C_k} A_{ij}$
        Compute $d_{\text{cut}}(P_i, C_k) = \sum_{P_j \notin C_k} A_{ij}$
        if $d_{\text{in}}(P_i, C_k) < \tau \cdot d_{\text{cut}}(P_i, C_k)$ **then**
          Remove P_i from C_k
        end if
      end if
      {Stage 2: Triangle-closure Expansion}
      if $P_i \notin C_k$ **then**
        Compute triangle support:
        $T(P_i, C_k) = |\{\{P_j, P_l\} \subseteq C_k : A_{ij} = 1,\ A_{il} = 1,\ A_{jl} = 1\}|$
        if $T(P_i, C_k) \geq \gamma$ **then**
          Add P_i to C_k
        end if
      end if
      {Stage 3: Triangle-support Pruning}
      if $P_i \in C_k$ **then**
        Compute internal triangle participation:

$$T_{\text{in}}(P_i, C_k)$$

        if $T_{\text{in}}(P_i, C_k) = 0$ **then**
          Remove P_i from C_k
        end if
      end if
    end if
  end for
end for
return $\mathcal{C}$
