# Supplementary material for: TOMOC: a topology-driven multi-objective evolutionary framework for robust overlapping protein complex detection in noisy PPI networks
Source: BMC Bioinformatics. 2026 Aug 18;27:189. doi: 10.1186/s12859-026-06603-z (PMC13488024; doi:10.1186/s12859-026-06603-z)
Supplement: Supplementary file 2 [file 12859_2026_6603_MOESM2_ESM.pdf]

Additional file 2: Extended Experimental Analyses

This supplementary file provides extended experimental analyses supporting the main manuscript. It includes additional comparisons with evolutionary and cut-based optimization methods on the Collins PPI network, an assessment of run-to-run stability, a characterization of the nondominated Pareto solutions, and supplementary robustness analyses under controlled interaction insertion and deletion. Unless otherwise stated, benchmark performance was evaluated using the matching threshold $OS = 0.20$.

S2.1 Comparison with cut-based genetic algorithms

To broaden the evaluation scope, we compared TOMOC with genetic algorithm-based methods proposed by Ramadan and Ahmed [5], which optimize classical graph cut objectives (Density cut, Maxmin cut, Normalized cut, and Ratio cut) using either spectral or random initialization.

Table S2.1: Performance comparison with cut-based genetic algorithms on the Collins network.

	CYC2008			MIPS		
**Algorithm**	Recall	Precision	F1-score	Recall	Precision	F1-score
Density cut$_{Spectral}$	0.7400	0.6000	0.6600	0.3200	0.4500	0.3700
Maxmin cut$_{Spectral}$	0.7100	0.4700	0.6000	0.3800	0.4000	0.3900
Normalized cut$_{Spectral}$	0.6700	0.5000	0.5700	0.3900	0.3700	0.3700
Ratio cut$_{Spectral}$	0.7300	0.3800	0.5000	0.3900	0.3300	0.3600
Density cut$_{Random}$	0.7200	0.5400	0.6200	0.3000	0.4100	0.3500
Maxmin cut$_{Random}$	0.7100	0.4500	0.5600	0.4000	0.3500	0.3800
Normalized cut$_{Random}$	0.6600	0.5700	0.6100	0.3600	0.4400	0.4100
Ratio cut$_{Random}$	0.6100	0.5500	0.5800	0.3400	0.4300	0.3800
**TOMOC**	**0.8690**	**0.8694**	**0.8690**	**0.8927**	**0.8549**	**0.8733**

S2.2 Comparison with canonical single-objective EAs

We further compared TOMOC with canonical evolutionary algorithms proposed by Pizzuti et al. [4], which optimize individual structural objectives such as community score, expansion, normalized cut, and modularity.

Table S2.2: Comparison with canonical single-objective EAs.

	CYC2008			MIPS		
**Algorithm**	Recall	Precision	F1-score	Recall	Precision	F1-score
EA-CS	0.7739	0.6313	0.6953	0.7718	0.4155	0.5401
EA-EX	0.7222	0.6234	0.6691	0.7335	0.4257	0.5386
EA-RC	0.7057	0.6276	0.6642	0.6988	0.4153	0.5209
EA-NC	0.6808	0.6233	0.6506	0.7004	0.4253	0.5292
EA-ID	0.7176	0.6149	0.6620	0.7387	0.4178	0.5336
EA-Q	0.7287	0.6338	0.6777	0.7218	0.4144	0.5263
**TOMOC**	**0.8690**	**0.8694**	**0.8690**	**0.8927**	**0.8549**	**0.8733**

S2.3 Comparison with heuristic-enhanced EAs

We also evaluated heuristic-enhanced EA variants proposed by Abbas et al. [1], which incorporate topology-aware mutation operators.

Table S2.3: Comparison with heuristic-enhanced evolutionary algorithms.

	CYC2008			MIPS		
**Algorithm**	Recall	Precision	F1-score	Recall	Precision	F1-score
EA-CS_{mu}	0.7954	0.6284	0.7020	0.7903	0.4194	0.5479
EA-EX_{mu}	0.7935	0.6265	0.7002	0.7714	0.4217	0.5452
EA-RC_{mu}	0.6571	0.6307	0.6435	0.6851	0.4220	0.5222
EA-NC_{mu}	0.6766	0.6352	0.6551	0.6806	0.4240	0.5225
EA-ID_{mu}	0.7444	0.6162	0.6741	0.7484	0.4167	0.5352
EA-Q_{mu}	0.6690	0.6296	0.6486	0.6931	0.4221	0.5246
**TOMOC**	**0.8690**	**0.8694**	**0.8690**	**0.8927**	**0.8549**	**0.8733**

S2.4 Comparison with multi-objective evolutionary algorithms

Finally, we compared TOMOC with two established multi-objective community-detection methods: $MOCD_1$, proposed by Bandyopadhyay et al. [3], and $MOCD_2$, proposed by Attea et al. [2].

Table S2.4: Comparison with multi-objective evolutionary algorithms.

	CYC2008			MIPS		
**Algorithm**	Recall	Precision	F1-score	Recall	Precision	F1-score
$MOCD_1$	0.8299	0.6393	0.7153	0.8177	0.4361	0.5655
$MOCD_2$	0.8326	0.6422	0.7165	0.8242	0.4338	0.5622
**TOMOC**	**0.8690**	**0.8694**	**0.8690**	**0.8927**	**0.8549**	**0.8733**

S2.5 Stability Analysis

To examine the run-to-run stability of TOMOC, we analyzed the F1-scores and the numbers of predicted complexes obtained across 10 independent runs. For each run, the representative solution was selected from the final nondominated archive according to the highest F1-score. The coefficient of variation was calculated as

$$\mathrm{CV}(\%) = 100 \times \frac{\mathrm{SD}}{\mathrm{Mean}} \tag{S2.1}$$

Table S2.5 reports the observed ranges and coefficients of variation. The F1-score coefficient of variation remained below 2% across all evaluated settings, indicating limited run-to-run variation in the reported benchmark performance. The number of predicted complexes showed moderately greater variability, indicating that different runs may produce complex sets of slightly different sizes while maintaining comparatively consistent F1-scores.

S2.6 Pareto-Front Characterization and Coupling with External Validation

A key component of TOMOC is its multi-objective formulation, which simultaneously optimizes average conductance (Obj1) and triangle-density loss

Table S2.5: Run-to-run stability of TOMOC across 10 independent runs using the Jaccard matching threshold denoted by $OS = 0.20$. CV denotes the coefficient of variation.

**Dataset**	**F1-score range**	**CV(F1) (%)**	**#Predicted range**	**CV(#Predicted) (%)**
Yeast-D1	0.868-0.908	1.62	146-165	3.95
Yeast-D2	0.817-0.856	1.36	189-223	5.46
Collins-CYC2008	0.854-0.879	0.92	294-338	4.18

(Obj2). Since both objectives are derived purely from network topology, we examined whether their values were associated with external agreement between the predicted and reference complexes.

For this analysis, all nondominated solutions contained in the final non-dominated archives were aggregated across the 10 independent runs for each dataset. For every solution, the external validation F1-score was calculated against the corresponding reference complex set using the matching threshold $OS = 0.20$. Spearman's rank correlation was then used to evaluate the monotonic associations between each objective and the external F1-score, as well as between Obj1 and Obj2.

This analysis examined two aspects: (i) whether the optimized topological objectives were associated with external benchmark agreement, and (ii) whether the nondominated solutions exhibited a trade-off between the two objectives.

For the Yeast-D1 dataset, a total of $N = 137$ nondominated solutions were collected across all runs and evaluated against 78 reference complexes under the applied matching criterion. Figure S2.1 presents the relationships between Obj1 and F1-score, Obj2 and F1-score, and Obj1 and Obj2.

Obj1 showed a positive association with F1-score ($\rho = 0.3827$, $p = 3.9 \times 10^{-6}$), whereas Obj2 showed a negative association with F1-score ($\rho = -0.4233$, $p = 2.5 \times 10^{-7}$). The direction of the Obj2 association is consistent with its formulation as a loss function, for which lower values represent lower triangle-density loss.

Obj1 and Obj2 also exhibited a strong negative association ($\rho = -0.7791$, $p < 0.001$), indicating that the two objectives captured competing structural characteristics among the nondominated solutions. These results suggest that triangle-based cohesiveness and conductance provide complementary topological information related to internal cohesion and boundary separation.

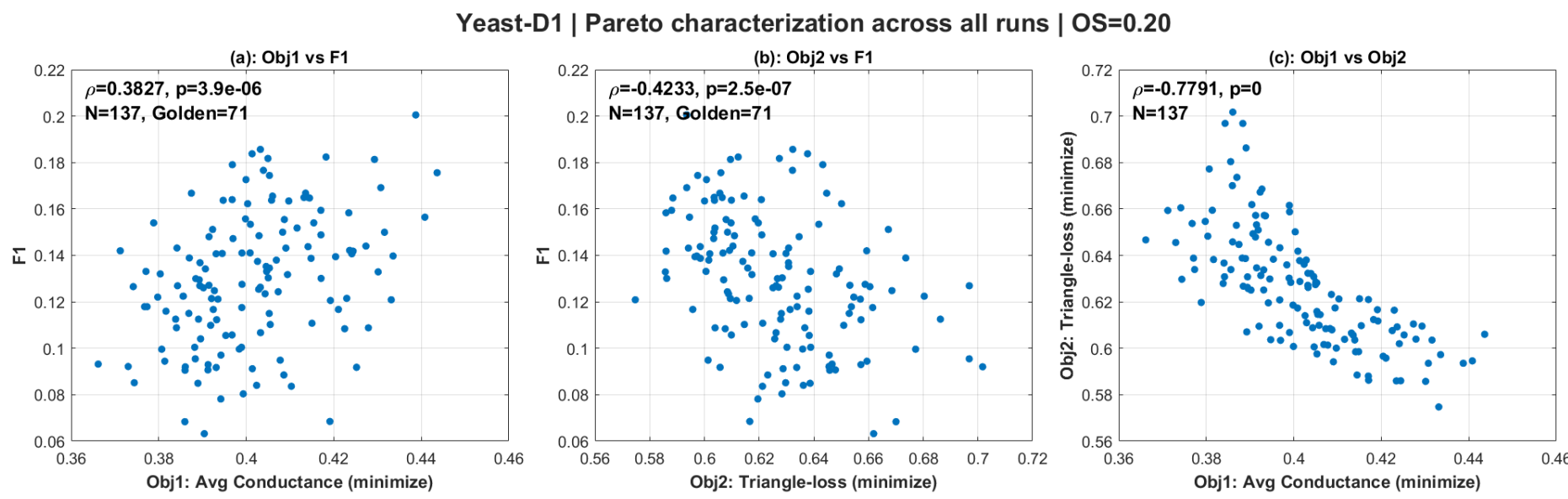

Figure S2.1: Pareto-front characterization on Yeast-D1 using $OS = 0.20$. (a) Obj1 versus F1-score, (b) Obj2 versus F1-score, and (c) Obj1 versus Obj2 across $N = 137$ nondominated solutions. Both objectives showed statistically significant associations with F1-score, while a strong negative association was observed between Obj1 and Obj2.

For Yeast-D2, a total of $N = 152$ nondominated solutions were evaluated against 150 reference complexes. As shown in Figure S2.2, Obj2 remained negatively associated with F1-score ($\rho = -0.3971$, $p = 4.1 \times 10^{-7}$), whereas Obj1 showed a weaker association that did not reach the conventional significance level ($\rho = 0.1542$, $p = 0.058$).

The association between Obj1 and Obj2 remained strongly negative ($\rho = -0.7522$, $p < 0.001$), indicating that the nondominated archive contained solutions representing different balances between conductance and triangle-density loss. Among the pooled Yeast-D2 solutions, triangle-density loss showed a stronger association with external F1-score than conductance.

For the Collins network, a total of $N = 146$ nondominated solutions were evaluated against 261 reference complexes. As shown in Figure S2.3, both objectives showed statistically significant, although weaker, associations with F1-score (Obj1: $\rho = 0.2104$, $p = 0.011$; Obj2: $\rho = -0.1757$, $p = 0.034$).

A strong negative association was observed between Obj1 and Obj2 ($\rho = -0.8533$, $p < 0.001$), indicating a pronounced structural trade-off among the nondominated solutions. The weaker objective-F1 associations show that the relationship between the optimized topological objectives and external benchmark agreement varied across the evaluated networks.

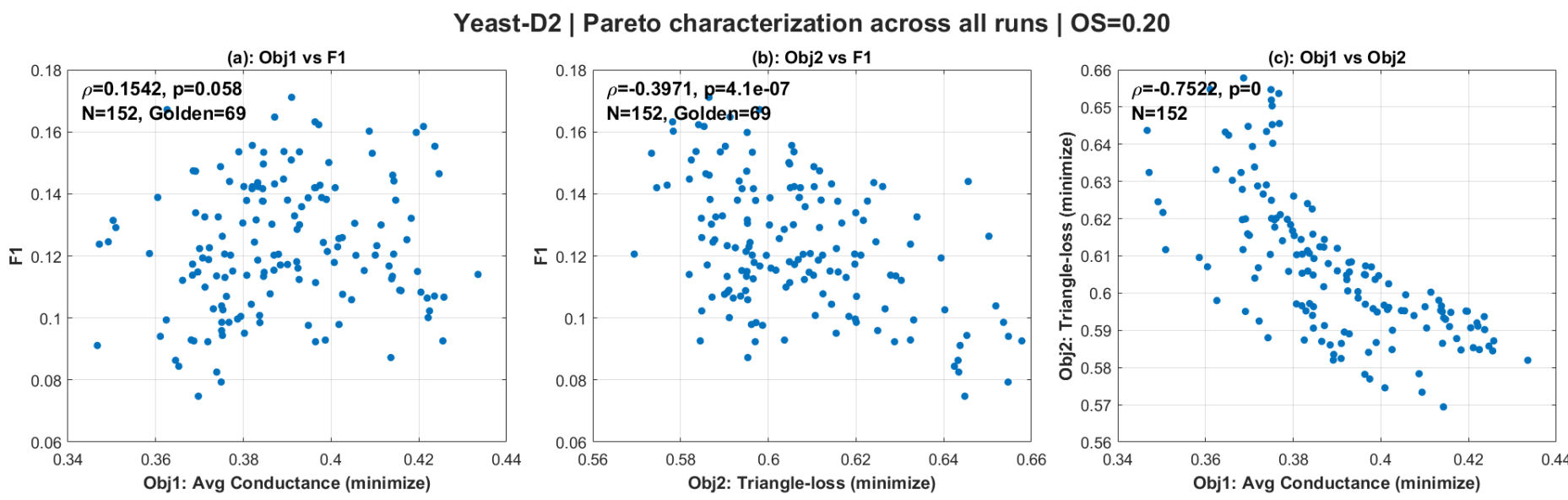

Figure S2.2: Pareto-front characterization on Yeast-D2 using $OS = 0.20$. (a) Obj1 versus F1-score, (b) Obj2 versus F1-score, and (c) Obj1 versus Obj2 across $N = 152$ nondominated solutions. Obj2 showed a stronger association with F1-score, while the two optimized objectives exhibited a strong negative association.

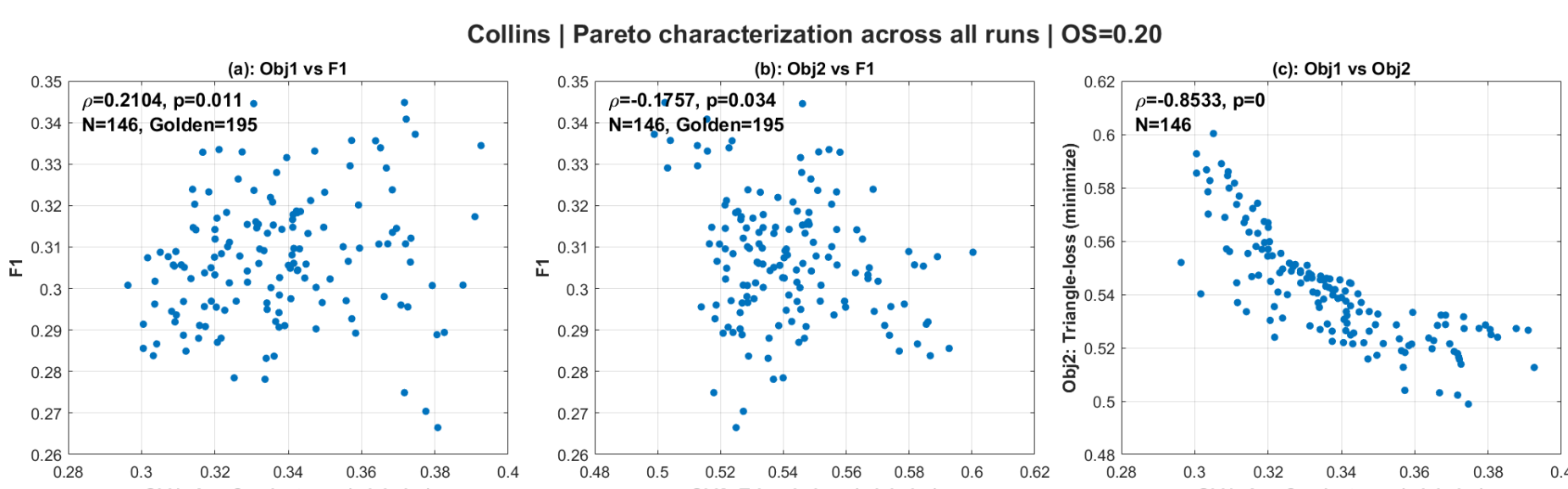

Figure S2.3: Pareto-front characterization on the Collins network using $OS = 0.20$. (a) Obj1 versus F1-score, (b) Obj2 versus F1-score, and (c) Obj1 versus Obj2 across $N = 146$ nondominated solutions. Both objectives showed comparatively weak associations with F1-score, while a strong negative association was observed between the two optimized objectives.

S2.7 Additional Robustness Analyses

The main manuscript presents the absolute F1-score curves obtained under controlled interaction insertion and deletion. This section provides additional summaries of the relative performance degradation across the evaluated perturbation scenarios.

The experiments comprised four evaluation settings: Yeast-D1, Yeast-D2, Collins-CYC2008, and Collins-MIPS. The latter two settings use the same Collins PPI network but different reference complex sets. Interaction insertion was used to simulate false-positive interactions, whereas interaction deletion was used to simulate missing interactions.

For each perturbation type, three interaction-selection strategies were considered: Random, Most, and Least. Random modifies interactions uniformly, whereas Most and Least prioritize interactions associated with high-degree and low-degree proteins, respectively. Perturbation levels ranged from 10% to 50% of the original number of interactions.

For every perturbation setting, TOMOC was executed over 10 independent runs. In each run, the representative solution was selected from the final nondominated archive according to the highest F1-score. Performance was evaluated using the matching threshold $OS = 0.20$.

The relative degradation in F1-score was calculated with respect to the performance obtained on the corresponding unperturbed network. As shown in Figure S2.4, interaction insertion generally produced greater degradation than interaction deletion. The largest reductions were commonly observed under RandomAdd and MostAdd, particularly at the higher perturbation levels. In contrast, deletion-based perturbations usually produced smaller changes in the reported F1-score.

Figure S2.5 provides an aggregated visualization of the Drop% values across perturbation levels and scenarios. The heatmaps highlight the comparatively greater influence of interaction insertion, particularly under RandomAdd and MostAdd. Deletion scenarios generally form a lower-degradation region across the evaluated settings.

Finally, Figure S2.6 summarizes the mean Drop% across the five perturbation levels for each scenario. This aggregated view shows the asymmetry between interaction insertion and deletion. Addition-based perturbations generally produced greater degradation, whereas deletion-based perturbations had a comparatively limited effect across the evaluated settings.

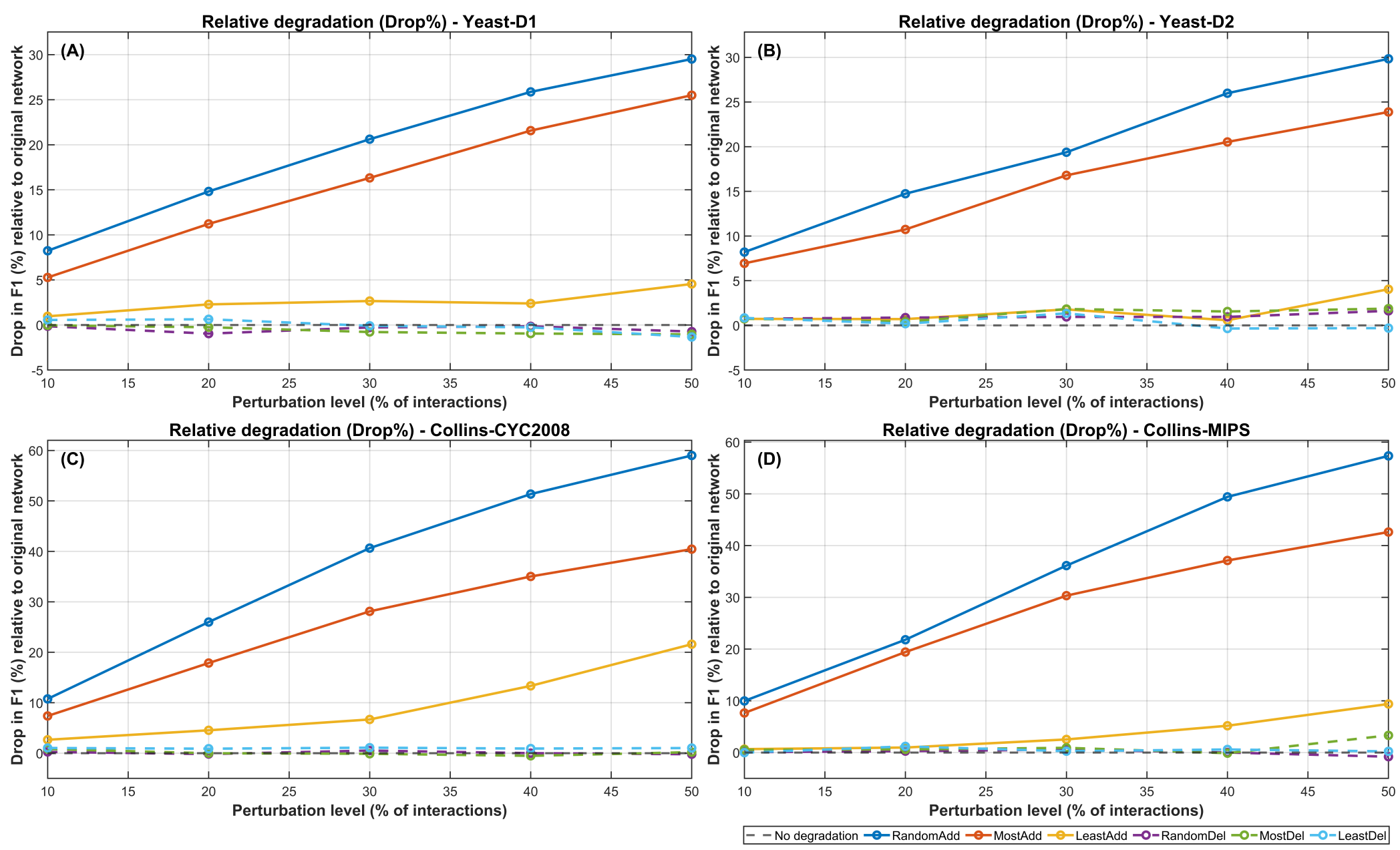

Figure S2.4: Relative F1-score degradation of TOMOC under controlled PPI-network perturbations. Drop% was calculated relative to performance on the corresponding unperturbed network using $OS = 0.20$. Interaction-addition scenarios (*RandomAdd*, *MostAdd*, and *LeastAdd*) and interaction-deletion scenarios (*RandomDel*, *MostDel*, and *LeastDel*) were evaluated at perturbation levels ranging from 10% to 50%. Panels correspond to (A) Yeast-D1, (B) Yeast-D2, (C) Collins-CYC2008, and (D) Collins-MIPS. The dashed horizontal line represents zero degradation.

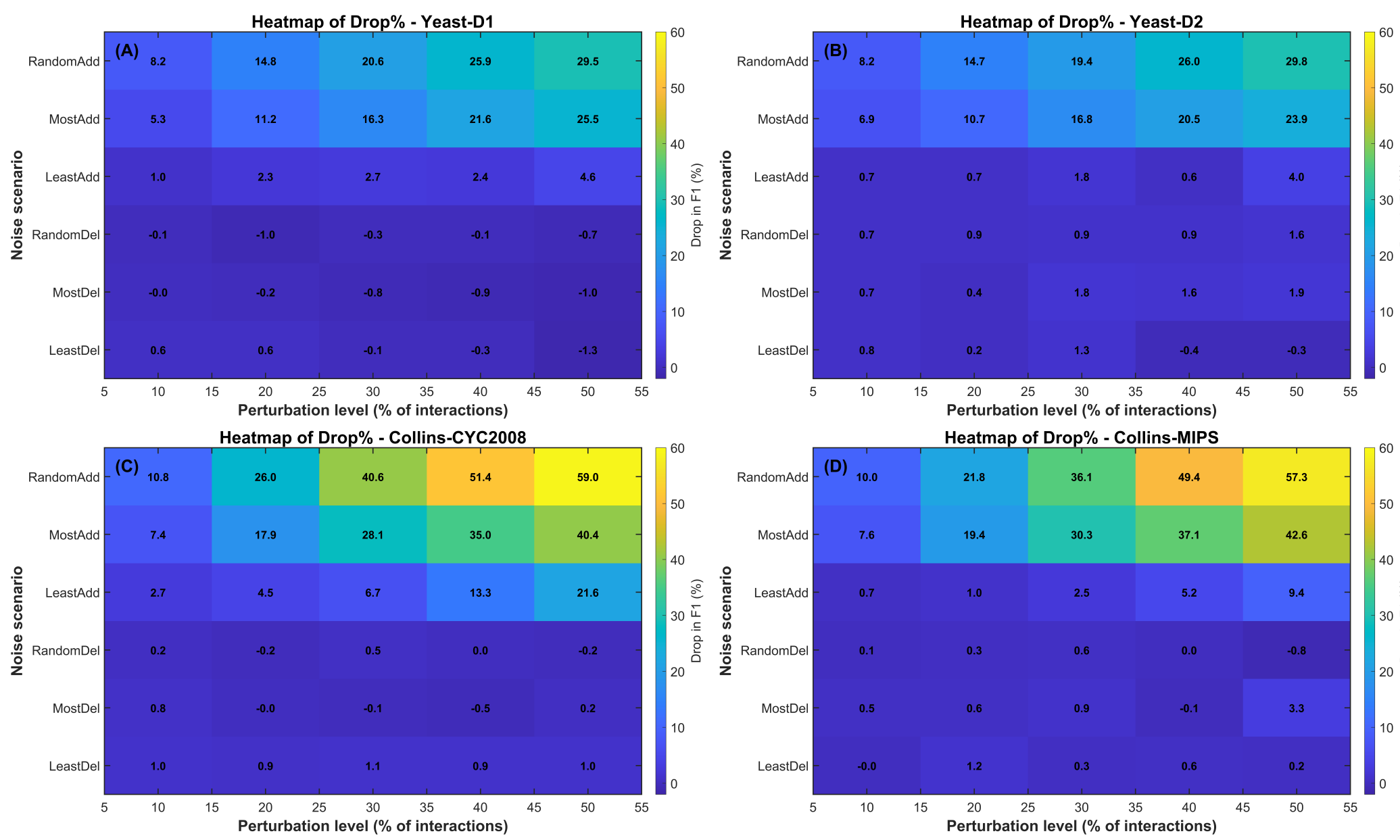

Figure S2.5: Heatmap summary of relative F1-score degradation across perturbation scenarios and levels using $OS = 0.20$. Rows represent the six perturbation scenarios, and columns represent perturbation levels from 10% to 50%. Each cell reports the Drop% relative to the corresponding unperturbed network. Panels correspond to (A) Yeast-D1, (B) Yeast-D2, (C) Collins-CYC2008, and (D) Collins-MIPS.

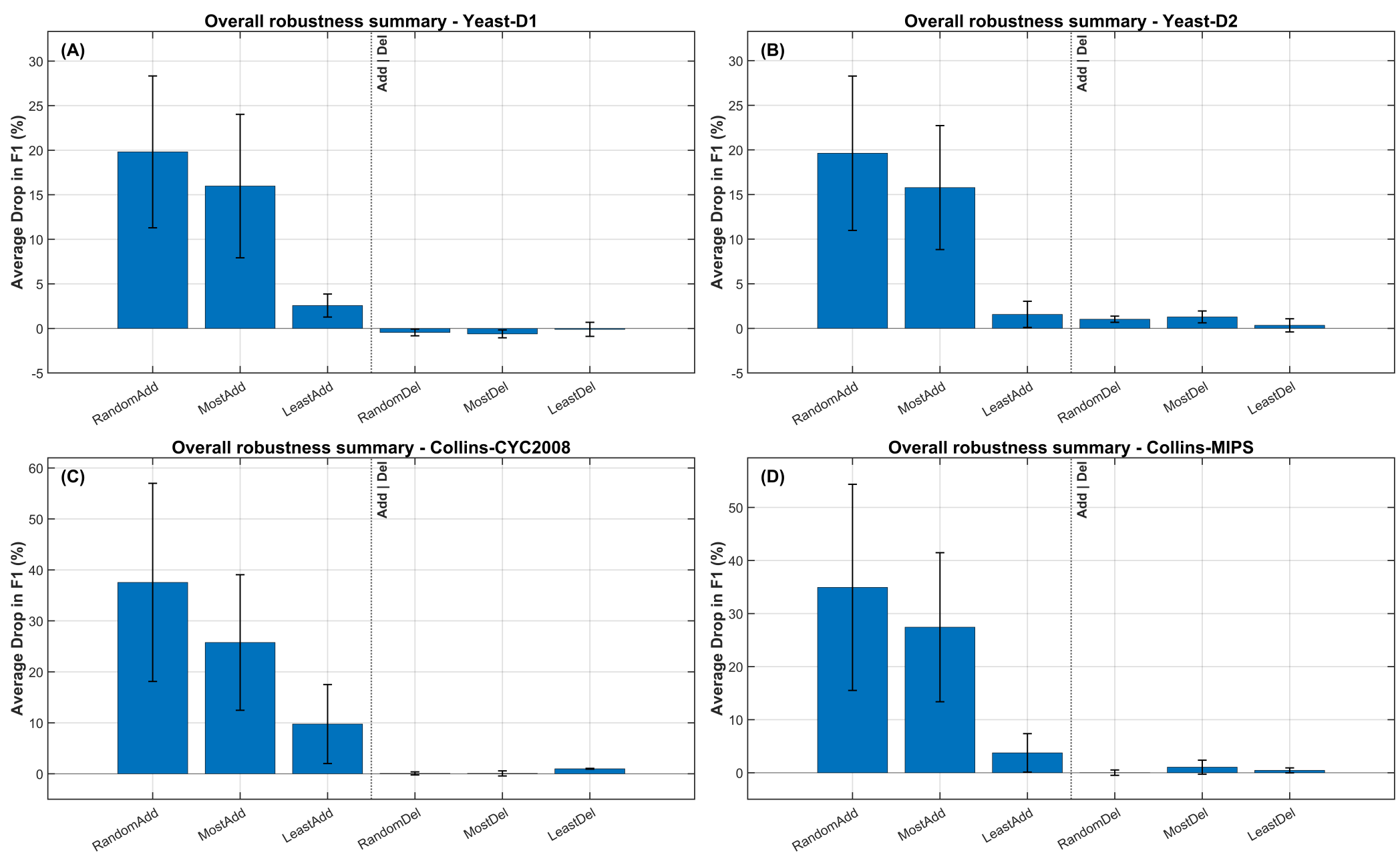

Figure S2.6: Average relative F1-score degradation across the five perturbation levels using $OS = 0.20$. For each evaluation setting, the bars represent the mean Drop% across perturbation levels from 10% to 50%, and the error bars represent variability across those levels. Panels correspond to (A) Yeast-D1, (B) Yeast-D2, (C) Collins-CYC2008, and (D) Collins-MIPS. The vertical separator distinguishes interaction-addition scenarios from interaction-deletion scenarios.

References

[1] Mustafa N Abbas, A Attea Bara'a, David Broneske, and Gunter Saake. An evolutionary algorithm with heuristic operator for detecting protein complexes in protein interaction networks with negative controls. *IEEE Access*, 2024.

[2] Bara'a A Attea and Qusay Z Abdullah. Improving the performance of evolutionary-based complex detection models in protein–protein interaction networks. *Soft Computing*, 22(11):3721–3744, 2018.

[3] Sanghamitra Bandyopadhyay, Sumanta Ray, Anirban Mukhopadhyay, and Ujjwal Maulik. A multiobjective approach for identifying protein complexes and studying their association in multiple disorders. *Algorithms for Molecular Biology*, 10(1):1–15, 2015.

[4] Clara Pizzuti and Simona E Rombo. Algorithms and tools for protein–protein interaction networks clustering, with a special focus on population-based stochastic methods. *Bioinformatics*, 30(10):1343–1352, 2014.

[5] Emad Ramadan, Ahmed Naef, and Moataz Ahmed. Protein complexes predictions within protein interaction networks using genetic algorithms. *BMC bioinformatics*, 17:481–489, 2016.
